# Use of High Energy Devices (HEDs) versus electrocautery for laparoscopic cholecystectomy: a systematic review and meta-analysis of randomised controlled trials

**DOI:** 10.1007/s00464-023-10060-7

**Published:** 2023-04-19

**Authors:** Monica Ortenzi, Ferdinando Agresta, Nereo Vettoretto, Chiara Gerardi, Eleonora Allocati, Emanuele Botteri, Giulia Montori, Andrea Balla, Alberto Arezzo, Giacomo Piatto, Alberto Sartori, Stavros Antoniou, Mauro Podda

**Affiliations:** 1 Department of General and Emergency Surgery, Polytechnic University of Marche, Ancona, Italy; 2 Unit of General and Emergency Surgery, Vittorio Veneto Hospital, Vittorio Veneto, Treviso Italy; 3 General Surgery, ASST Spedali Civili Di Brescia PO Montichiari, Montichiari, Brescia Italy; 4 Istituto Di Ricerche Farmacologiche Mario Negri IRCCS, Milano, Italy; 5 General and Minimally Invasive Surgery, Hospital “San Paolo”, Civitavecchia, Rome Italy; 6 Department of Surgical Sciences, University of Torino, Turin, Italy; 7 Department of General Surgery, Ospedale Di Montebelluna, Montebelluna, Treviso Italy; 8 Department of Surgery, Mediterranean Hospital of Cyprus, Limassol, Cyprus; 9 Department of Surgical Science, Emergency Surgery Unit, University of Cagliari, Cagliari, Italy

**Keywords:** High Energy Devices, Cholecystectomy, Laparosocpic surgery

## Abstract

**Introduction:**

According to the literature, there is no clear definition of a High Energy Devices (HEDs), and their proper indications for use are also unclear. Nevertheless, the flourishing market of HEDs could make their choice in daily clinical practice arduous, possibly increasing the risk of improper use for a lack of specific training. At the same time, the diffusion of HEDs impacts the economic asset of the healthcare systems. This study aims to assess the efficacy and safety of HEDs compared to electrocautery devices while performing laparoscopic cholecystectomy (LC).

**Materials and methods:**

On behalf of the Italian Society of Endoscopic Surgery and New Technologies, experts performed a systematic review and meta-analysis and synthesised the evidence assessing the efficacy and safety of HEDs compared to electrocautery devices while performing laparoscopic cholecystectomy (LC). Only randomised controlled trials (RCTs) and comparative observational studies were included. Outcomes were: operating time, bleeding, intra-operative and post-operative complications, length of hospital stay, costs, and exposition to surgical smoke. The review was registered on PROSPERO (CRD42021250447).

**Results:**

Twenty-six studies were included: 21 RCTs, one prospective parallel arm comparative non-RCT, and one retrospective cohort study, while three were prospective comparative studies. Most of the studies included laparoscopic cholecystectomy performed in an elective setting. All the studies but three analysed the outcomes deriving from the utilisation of US sources of energy compared to electrocautery. Operative time was significantly shorter in the HED group compared to the electrocautery group (15 studies, 1938 patients; SMD − 1.33; 95% CI − 1.89 to 0.78; I2 = 97%, Random-effect). No other statistically significant differences were found in the other examined variables.

**Conclusions:**

HEDs seem to have a superiority over Electrocautery while performing LC in terms of operative time, while no difference was observed in terms of length of hospitalisation and blood loss. No concerns about safety were raised.

**Supplementary Information:**

The online version contains supplementary material available at 10.1007/s00464-023-10060-7.

High Energy Devices (HEDs) gave substantial input to the development of modern surgery [1]. The introduction of HEDs was almost contemporary with the laparoscopic revolution. Together, they made vessel sealing, coagulation, and transection much more efficient and safer than in the past, reducing operative time and improving surgical proficiency [1–3].

However, assessing the contribution of HEDs to laparoscopy may not be so intuitive. As a result, the topic is often overlooked in guidelines or only shortly mentioned. Moreover, the literature about HEDs in different surgical settings is too scarce and inconsistent to allow an explicit agreement on whether HEDs should be used and which is the ideal HED in a specific surgical setting [4] or their added value.

Laparoscopic cholecystectomy (LC) is one of the most performed procedures worldwide at any level of surgical expertise, both in elective and emergency settings. The implementation of HEDs in this procedure is controversial for many reasons, ranging from the economic impact to the surgeon’s personal experience [4–7]. This study aimed to assess the efficacy and safety of HEDs compared to electrocautery devices in LC through a systematic review and meta-analysis of the available literature.

## Methods

This systematic review and meta-analysis are part of a series of syntheses of the evidence performed to assess the efficacy and safety of HEDs in surgery in terms of Health Technology Assessment. It was reported according to the recommendations of the 2020 updated Preferred Reporting Items for Systematic reviews and Meta-analyses (PRISMA) statement [8] and conducted in line with the Cochrane handbook for systematic reviews of interventions [9].

The PICO questions were generated as the results of a discussion within a commission of clinicians from the Italian Society of Endoscopic Surgery and new technologies (SICE—Società Italiana di Chirurgia Endoscopica e nuove tecnologie), methodologists from the Mario Negri Institute for Pharmacological Research, clinical engineers from the Polytechnic of Milan, and economists from the LIUC—Carlo Cattaneo University of Castellanza, Italy. Institutional review board approval was not required for this study.

The following PICO question was adopted:

- P(opulation). Patients (age ≥ 18) undergoing laparoscopic or open cholecystectomy.
- I(ntervention). High energy devices (HED): ultrasonic (US), radiofrequency (RF), and hybrid US/RF energy (H-US/RF) devices.
- C(omparison). Monopolar or bipolar devices.
- O(utcomes). Operating time, bleeding, intra-operative and post-operative complications (outcomes of safety and efficacy); other outcomes (length of hospital stay, costs, and exposition to surgical smoke).

### Study identification

A computerised search was performed in MEDLINE (via PubMed), EMBASE and the Cochrane Central Register of Controlled Trials databases for articles published from the inception to 30/07/2022, with no language or publication type restrictions. Search terms included extensive controlled vocabulary (MeSH and EMTREE) and free-text keywords, combining the conditions (e.g. laparoscopic cholecystectomy, gallbladder surgery, etc.), interventions (high energy device, ultrasonic, radiofrequency, etc.), and control group (e.g. monopolar electrosurgery, bipolar electrosurgery, etc.). Details on the search strategies can be found in supplementary data (Supp Box 1) and PROSPERO (CRD42021250447). We checked the reference lists of relevant studies to retrieve further studies and congress abstracts and searched study registries for unpublished or ongoing studies.

### Eligibility criteria, screening process and data extraction

Randomised controlled trials (RCTs) and comparative observational studies (both prospective and retrospective) that compared HEDs and monopolar or bipolar devices in the setting of cholecystectomy were eligible for inclusion in the present systematic review and meta-analysis. Two independent reviewers conducted the screening process and data extraction (AS and GP) in a double-blind fashion. Discrepancies were resolved with a discussion with a third reviewer (MO).

### Types of studies

**Fig. 1:**
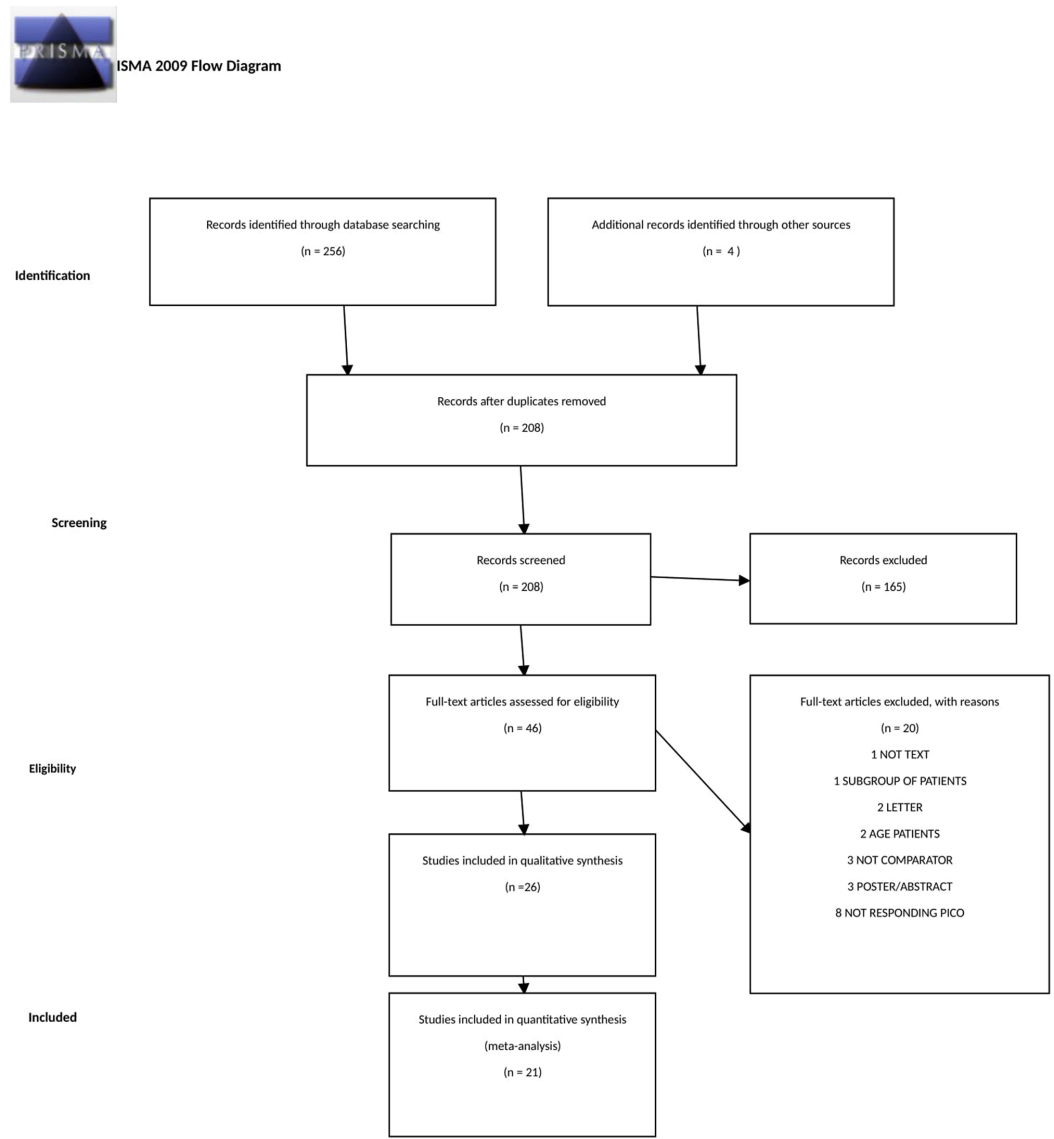
PRISMA flow diagram

This systematic review and meta-analysis included 26 studies dealing with the use of HEDs: ultrasonic (US), radiofrequency (RF), and hybrid US/RF energy (H-US/RF) in the setting of cholecystectomy. Twenty-one studies were RCTs that compared different HEDs to monopolar energy. All the studies but two compared two study groups (the US *vs* electrocautery), whereas Wetter et al*.* [10] *and Bulus *et al*.* [11] included three groups (Laser, CUSA and electrocautery; US, RF and electrocautery, respectively) (Fig. 1) Five studies were observational cohort studies (Bessa 2008, Zanghì 2014, Schulze 2010, Rajinish 2018) and one was retrospective (Gelmini 2010). The others were prospective [12–16] (Table 2).

### Participants

All the papers included adult patients (≥ 18 years old) undergoing LC conducted with HED. Wide variability was found concerning indications to cholecystectomy: symptomatic gallstones, acalculous cholecystitis, acute and chronic cholecystitis, and gallbladder polyps (Table 3). No restriction based on the type of anaesthesia or patient positioning during the surgical intervention was applied, reporting that the same kind of anaesthesia was used in both groups.

### Interventions and comparators

Twenty-three studies compared US vs monopolar [12–14, 16–34], and one compared RF vs monopolar [15]. No studies were comparing H-US/RF vs monopolar. Two RCTs compared three groups (Table 1) [10, 11].

**Table 1:** Characteristics of RCTs included in the systematic review

Author (year) [ref]	Country	Duration of study	N of randomized Pts (pts include in the study)	Setting	Study arms		N of Pts for arm	
					Study	Control	Study	Control
Wetter A.L. (1992) [10]	USA	Jan 1991 – May 1991	40	Elective	Laser/CUSA	Electrocautery	52 15 Laser 37 CUSA	21
Tsimoyiannis E.C. (1998) [17]	Greece	NA	200	Elective	US	Electrocautery	100	100
Sietses C. (2000) [18]	Netherlands	NR	18	Elective	US	Electrocautery	9	9
Janssen I.M.C. (2003) [19]	Netherlands	June 1998 – Jan 2000	199	Elective	US	Electrocautery	96	103
Cenzig Y(2005) [20]	Sweden	June 2002 – March 2004	80	Elective/ Acute cholecystitis	US	Electrocautery	37	43
Cenzig Y (2009) [21]	Sweden	Oct 2006 – Oct 2007	243 (233)	Elective/ Acute cholecystitis	US (fundus-first)	Electrocautery	77	166 (85 fundus-first)
Kandil T. (2010) [22]	Egypt	Jan 2008- Dec 2008	140	NR	US	Electrocautery	70	70
El Nakeeb A. (2010) [23]	Egypt	Aug 2009- Oct 2009	120	Elective/ Acute cholecystitis	US	Electrocautery	60	60
Redwan A.A. (2010) [24]	Egypt	Jan 2008- July 2009	160	NR	US	Electrocautery	80	80
Mahabaleshwar V.(2011) [25]	India	July 2008- Dec 2009	60	NR	US	Electrocautery	30	30
Jain S. K. (2011) [26]	India	NR	200 (192)	Elective	US	Electrocautery	96	96
Tempè F. (2013) [27]	Sweden	June 2002- March 2004	80 (73)	Elective	US	Electrocautery	40 (fundus-first)	33
Bulus H. (2013) [11]	Turkey	July 2010- Jan 2011	60	Consecutive patients	US/RF	Electrocautery	20/20	20
Ramzanali (2013) [28]	Pakistan		92	Elective	US	Electrocautery	46	46
Catena F. (2014) [29]	Italy	NR	42	Acute cholecystitis	US	Electrocautery	21	21
Sista F (2014) [30]	Italy	May 2005- May 2012	43	Acute cholecystitis	US	Electrocautery	22	21
Baloch S.H. (2015) [31]	Pakistan	Jan 2014- Jan 2015	86	Elective	US	Electrocautery	43	43
Liao G. (2016) [32]	China	Oct 2010- June 2013	234 (198)	Elective/ Acute cholecystitis	US	Electrocautery	117	81
Shabbir A. (2016) [33]	Pakistan	Jan 2015- Dec 2015	120	NR	US	Electrocautery	60	60
Ahmed A.(2019) [34]	Pakistan	June 2016- August 2018	144	Elective	US	Electrocautery	72	72

*Pts* patients, *ref* reference, *US* Ultrasonic, *RF* Radiofrequency

### Outcomes of interest

According to the PICO criteria, we included general and clinical primary outcomes into the analysis: operative time, intraoperative bleeding, intra-operative complications, post-operative complications and length of stay. These outcomes of interest were entered in the pooled analysis. Secondary outcomes were the length of hospital stay, quality of life, including patients’ reported outcomes, and production of surgical smoke.

### Study selection and data extraction

Two reviewers independently screened titles and abstracts to select the studies (AS, GP). One reviewer reviewed the full-text publication to confirm the eligibility and extract the relevant information from the included trials (GP). A second reviewer checked the eligibility and the data extraction to increase the accuracy of the process (AS). Any discrepancies were resolved by consensus and arbitration by a third author. Data collected from each study included the following predefined items: (1) Study identifier (first author, year of publication); (2) Reference; (3) Other publication; (4) Study design; (5) Population; (6) Study duration; (7) Follow-up; (8) Sample size; (9) Intervention/control group (10) Outcome measure; (11) Main results; (12) Conclusion; (13) Risk of bias/quality assessment. A predefined spreadsheet (Excel 2007, Microsoft Corporation®) was used for data extraction.

### Data synthesis, analysis and assessment of heterogeneity

All statistical analyses were performed using Reviewer Manager software (Reviewer Manager—RevMan—version 5.4.1, Sept. 2020, The Nordic Cochrane Centre, Cochrane Collaboration, www.training.cochrane.org). The risk ratio (RR) with a 95% confidence interval (95% CI) was calculated for dichotomous variables, and the weighted standardised means difference (SMD) with 95% CI for continuous variables. Whenever continuous data were reported as medians and ranges, the method of Hozo et al. to estimate respective means and standard deviations was applied [35]. Unless stated otherwise, statistical significance was assessed at the two-sided 5% significance level. Statistical heterogeneity of the results across studies was assessed using the Higgins’ *I*^*2*^ and Chi-Square test. A *P* value of the Chi-Square test < 0.10 with an *I*^*2*^ value > 50% indicated substantial heterogeneity. Besides statistical heterogeneity, we considered both clinical (variability in the baseline characteristics of the participants, interventions and outcomes studied) and methodological (variability in the study design and risk of bias) heterogeneity to inform the decision to use the random-effects model. Funnel plots were constructed to visually detect the risk of publication bias and any association between treatment estimated and sample size, in keeping with the recommendations by the Cochrane Collaboration.

Sensitivity analyses on clinically relevant outcomes were performed based on the qualitative evaluation of the included studies. Given that substantial differences in clinical settings among individual studies were expected, a subgroup analysis focused on the primary outcome of HEDs and Electrocautery in patients requiring cholecystectomy for acute cholecystitis was planned.

### Risk of bias assessment in the included studies

The risk of bias in the included RCTs was independently assessed by two authors using the Cochrane Risk of Bias 2.0 (RoB 2.0) tool without masking the trial names. The methodological quality of the RCTs was assessed based on the randomisation process, deviations from the intended intervention, missing outcome data, measurement of the outcome, and selection of the reported results. Trials classified as low risk of bias in the randomisation process, deviations from intended intervention, missing outcome data, measurement of the outcome, and selection of the reported results were judged at low bias risk following a given algorithm. The risk of bias in observational studies was assessed using the ROBINS-I tool. A detailed risk of bias judgement was provided together with a summary using the *robvis* tool.

### Grading the quality of evidence

According to the GRADE approach, two authors independently evaluated the quality of evidence for imprecision, inconsistency, indirectness, and publication bias. Further, the quality of evidence was classified as very low, low, moderate, or high [36, 37]. Subsequently, a summary table was created using GRADE profiler software (version 3.6.1) (available at: https://www.gradeworkinggroup.org/).

## Results

The initial search produced 256 potentially relevant articles. After removing duplicates, 208 abstracts were assessed for eligibility, and only 46 full texts were admitted to subsequent assessments. Twenty-six studies were included in the systematic review after full-text evaluation. Details of the screening process, including reasons for full-texts exclusion, are reported in Fig. 1 (PRISMA flow-chart). Of 26 included studies, 21 were RCTs, one was a prospective parallel arm comparative non-randomised trial (Rajinish 2018), one (Gelmini 2010) was a retrospective cohort study, and three were prospective comparative studies (Bessa 2008, Schulze 2010, Zanghì 2014). Most of the studies included LC performed in an elective setting, while the setting was not specified in five studies (Kandil 2010, Redwan 2010, Mahabaleshwar 2011, Bulus 2013, Shabbir 2016). Six studies did not consider acute cholecystitis an exclusion criterion (Kandil 2010, Redwan 2010, Gelmini 2010, Cengiz 2009, El Nakeeb 2010, Liao 2016). Two studies (Catena 2014, Sista 2015) specifically addressed patients with acute cholecystitis, with or without peritonitis. In another study (Cenzig 2005), the authors specifically analysed the differences in outcomes between patients with and without cholecystitis. All the studies but three (Wetter 1992, Bulus 2013, Schulze 2010) analysed the outcomes deriving from the utilisation of US sources of energy compared to electrocautery. HEDs were used to close the cystic duct and the artery without the application of clips in eight studies (Redwan 2010, Gelmini 2010, Zanghì 2014, Baloch 2015, Cengiz 2005, Kandil 2010, Schulze 2010, Bulus 2013). Bulus et al. [11] and Wetter et al. [10] compared three groups according to the type of the HED used: Group A = electrocautery, Group B = Harmonic scalpel, Group C = Bipolar vessel sealer and Laser, CUSA and electrocautery, respectively.

Schulze et al. [15] analysed the outcomes derived from the utilisation of RF used for both dissection and closure of the cystic duct and the artery. In two studies, a fundus-first technique was described in addition to using HED (Tempè 2013) or as a comparator technique in the control group (Cenzig 2009). Table 1 and Table 2 summarise the characteristics of the included studies.

**Table 2:** Characteristics of the cohort observational studies included in the systematic review

Author (year) [ref]	Country	Period of interest	N of pts included in the study	Setting	Study groups		N of Pts for gropus	
					Study	Comparator	Study	Comparator
Gelmini R. (2010) [13]	Italy	2 years (retrospective)	185	Consecutive patients	US	Electrocautery	95	90
Zanghì A. (2014) [14]	Italy	Jan 2019-Dec 2011 (prospective)	164	Consecutive patients	US	Electrocautery	43	121
Schulze S. (2010) [15]	Denmark	Jan 2007- Jan 2008 (prospective)	218 (215)	Elective	RF	Electrocautery	101	113
Rajinish K. (2018) [16]	India	NR Prospective, Parallel arms non RCT	40	Elective	US	Electrocautery	20	20
Bessa S.S (2008) [12]	Egypt	NR Prospective	120	Elective	US	Electrocautery	60	60

*Pts* patients, *N* number, *ref* reference, *NR* Not reported, *RCT* Randomized Controlled Trial

### Qualitative analysis

Table 3 and Table 4 summarise the characteristics of the populations in the included RCTs and observational studies, respectively.

**Table 3:** Patients characteristics of the included RCTs with more than 2 study groups

Author (year) [ref]	Study Arms	Mean/median age in yrs	Male (%)	Mean/Median BMI in kg/m^2^	Comorbidities	Cholecystitis N (%of pts)	Surgeon’s experience
Wetter A.L. (1992) [10]	Laser	44.4*	4 (36.4)	177*	NR	/	NR
	CUSA	48.8*	7 (23.3)	153.6*			
	Electrocautery	49.9*	6 (28.6)	162.8*			
Tsimoyiannis E.C. (1998) [17]	US	NA	NA	NA	NA	/	NA
	Electrocautery						
Sietses C. (2000) [18]	US	49.5* ± 14.1	NR	NR	NR	/	NR
	Electrocautery	53.3* ± 14.1					
Janssen I.M.C. (2003) [19]	US	50.0** (18–82)	78 (81.2)	25.5** (18.7–43.0)	NR	/	< 10 procedures 23 10–20 procedures 10 > 20 procedures 63
	Electrocautery	52.0** (21–85)	75 (72.8)	26.6** (17.9–42.5)	NR	/	< 10 procedures 12 10–20 procedures 18 > 20 procedures 73
Cenzig Y(2005) [20]	US	46** (40, 47)	13 (30.3)	27** (95% CI 26, 29)	NR	19 (43)	2 expert surgeons
	Electrocautery	44** (43, 50)	7 (18.9)	27** (95% CI 25, 28)		19 (37)	
Cenzig Y (2009) [21]	US	45** (42–48)	22 (27.5)	27** (95% CI 26–28)	NR	24 (33)	at least 15 procedures with each method
	Fundus-first Electrocautery	49** (45–52)	26 (30.9)	27** (95%CI 26–28)		26 (32)	
	Conventional Electocautery	47** (44–50)	16 (21.3)	27** (95%CI 26–28)		16 (20)	
Kandil T. (2010) [22]	US	40.97* ± 11.56	29 (41.4)	28.14* ± 3.87	Pre-existing comorbidities reported	NR	
	Electrocautery	41.38* ± 11.91	30 (42.8)	28.64* ± 4.46			
El Nakeeb A. (2010) [23]	US	41.42* ± 10.36	42 (70)	NR	Pre-existing comorbidities reported (patients with compensated cirrhosis)	6 (10)	Experienced
	Electrocautery	39.93* ± 13.82	35 (58.3)			4 (6.7)	
Redwan A.A. (2010) [24]	US	Pts divided in age groups no means provided	27 (33.7)	NR	NR	NR	NR
	Electrocautery		33 (41.2)				
Mahabaleshwar (2011) [25]	US	45.3*	1:2.75 M:F ratio	27.53*	Pre-existing comorbidities reported	2 wall > 3 mm	2 experienced surgeons
	Electrocautery	47.36*	1:1.5 M:F ratio	26.38*		7 wall > 3 mm	
Jain S. K. (2011) [26]	US	39.55* ± 11.12	6.25	NR	NR	/	NR
	Electrocautery	38.67** ± 11.87	11.4				
Tempè F. (2013) [27]	US	43.2 (11.8)	43.3	27.1 (4.4)	NR	16 (40)	NR
	Electrocautery	45.8 (11.6)	2.3	26.2 (2.9)		15 (45.4)	
Bulus H. (2013) [11]	US	NR	NR	NR	NR	0	NR
	RF						
	Electrocautery						
Ramzanali (2013) [28]	US	40.04* ± 7.84	7 (7.6)	NR	30 (32.6) ASA-2 62 (67.4) ASA-1	/	Experienced (more than 5 years post fellowship experience)
	Electrocautery						
Catena F. (2014) [29]	US	71.2* ± 7.1	11(52)	26.6* ± 2.1	NR	42	Experienced
	Electrocautery	71.6 * ± 6.2	10 (47)	28.1* ± 2.31			
Sista F (2014) [30]	US	40.04 ± 7.84	7 (7.6)	NR	ASA I 66 (68.7) ASA II 30 (32.6)	46	Experienced (more than 5 years post fellowship experience)
	Electrocautery					46	
Baloch S.H. (2015) [31]	US	43.62* (24 – 71)	3 (7.3)	NR	NR	/	NR
	Electrocautery	44.19*(23 – 71)	4 (10.0)				
Liao G. (2016) [32]	US	42.2* ± 10.4	51/66	24.6* ± 3.1	Pre-existing comorbidities reported	0	> 1000 conventional LC, 300 LC with HS
	Electrocautery	43.4 * ± 11.1	40/41	25.0* ± 3.6			
Shabbir A. (2016) [33]	US	35.88* ± 6.52	19(31.67)	NR	NR	NR	NR
	Electrocautery	36.41* ± 6.24	16(26.67)				
Ahmed (2019) [34]	US	34.5* ± 8.48	14 (19.4)	NR	NR	/	NR
	Electrocautery	36.25* ± 7.64	20 (27.8)				

*Pts* patients, *N* number, yrs years, *BMI* body mass index *NR* not reported, ^#^ Trendelenburg position, *LC* laparoscopic cholecystectomy, *US* Ultrasonic, *NR* Not Reported, *NA* Not available

^*^ ± mean ± Standard deviation; **median

**Table 4:** Patients characteristics of the included observational studies

Author (year) [ref]	Study Arms	Mean/Median age in yrs	Male (%)	Mean BMI in kg/m^2^ (SD)	Comorbidities	Cholecystitis *N* (%of pts)	Surgeon’s experience
Bessa S.S (2008) [12]	US	41.5* ± 10.3	13 (21.7)	31.2* ± 3.50	Pre-existing comorbidities reported	0	at least 100 successful laparoscopic cholecystectomies
	Electrocautery	42.56 * ± 11.4	12 (20)	31* ± 4			
Gelmini R. (2010) [13]	US	51.08 * ± 16.41	37(38.95)	NR	NR	13 (13.68%)	
	Electrocautery	52.05 * ± 18.13	37 (41.11)			15 (16.67%)	
Zanghì A. (2014) [14]	US	NR	NR	NR	NR		
	Electrocautery						
Schulze S. (2010) [15]	RF	32** (range 19–74)	32(31.7)	NR	NR	0	Experienced
	Electrocautery	39** (range 20–71)	39(				
Rajinish K.(2018) [16]	US	40.25* ± 14.85	6(30)	23.55* ± 4.75	ASA l 12(60) ASA ll 8 (40)	0	NR
	Electrocautery	46.6* ± 11.39	9(45)	23.62* ± 4.22	ASA l 11(55) ASA ll 9(45)		

*Pts* patients, *N* number, *yrs* years, *SD* standard deviation, *BMI* body mass index, *ASA* American Society of Anesthesiologists, *NR*: not reported

^*^ ± mean ± standard deviation, **median

### Operative time

Was analysed in all the included studies. All the included RCTs, but five reported a significantly reduced operative time when HEDs were used (Table 5). In Kandil et al. [22], the mean operative time was significantly shorter in the HED group than in the traditional group (33.21 ± 9.62 min vs 51.7 ± 13.79, p = 0.0001). In El Nakeeb et al. [23], the operative times were respectively 45.17 ± 10.54 in the US group and 69.71 ± 13.01 (30–90) in the electrocautery group (*p* = 0.000). In one study, the authors reported a time of approximately 2–3 min to dissect the cystic duct depending on the ductal thickness and associated inflammation, and operative time was significantly lower in the US group (16.8 ± 6.8 *vs* 44.01 ± 6.47, *p* = 0.0001) [24]. Mahabaleshwar et al. [25] reported a mean operative time of 27.20 min in the US group and 34.37 min in the control group (*p* = 0.001), while Jain et al. [26] reported an operative time of 50.00 ± 9.356 min in the US group *vs* 64.70 ± 13.74 min in the control group (*p* = 0.001). In Ramzanali et al. [24], the mean operative time was halved when the HED was used. Baloch et al. [31] reported a mean operative time of 21.55 min (range 12 – 38 min) in the US group compared to a mean time of 26.63 min (range 15–44 min) in the control group (*p* = 0.002). Bulus et al. [11] reported a duration of surgery of 31.5 ± 11.1 min in the US group, 33.1 ± 10 min in the electrocautery group, and 36.5 ± 9.9 in the RF group, and the difference between the US and RF groups was statistically significant (*p* < 0.04). Wetter et al. [10] reported a mean operative time of 81 min, without a significant difference between CUSA and electrocautery (90 vs 97 min), while the procedures were shorter when the laser was used (56 min). In two studies (Cenzig 2005, Tempè 2013), a fundus-first technique was adopted in association with the utilisation of a HED, and in both studies, operative time was significantly shorted when the HED was used. Cengiz et al. [20] identified two subgroups of patients according to the presence or absence of cholecystitis. They found out that the presence of cholecystitis prolonged the operation significantly when dissection was performed with electrocautery. Sista et al. [30] reported a significantly lower operative time (70.1 min IQR 51–115 *vs* 55.2 min IQR 39–90, *p* < 0.05) in patients with acute cholecystitis and LC performed as soon as possible, within 12 h of admission. In four papers, the operative time was shorter but not statistically different between the two groups (Siestes 2000; Janssen 2003; Catena 2014; Rajnish 2018), whereas, in one study (Liao 2016), the operative time was longer in the HED group, although the difference did not reach a statistical significance (54.9 ± 13.1 *vs* 51.7 ± 9.6, *p* = 0.079). Shabbir et al. reported shorter operative times in the HED group without providing a statical significance analysis. Among the observational studies, the operative time in minutes in the HED group ranged from a mean of 31.97 ± 4.34 min (Shabbir 2016) to a median of 60 min (IQR 20–205) (Gelmini 2010) and from a mean of 33.1 ± 10 min (Zanghì 2014) to a median of 85 min (range 45–150) (Gelmini 2010) in the electrocautery group. In one study (Schulze 2010), the authors did not report operative time. Bessa et al. [12] reported a statistically significant shorter median operative in the US group compared to the electrocautery group (32 vs 40 min, *p* = 0.000). The statistical significance was maintained in the absence of gallbladder perforation (mean 32.1 ± 7.6 *vs* 36.1 ± 6.2 min, *p* = 0.009). However, in the case of gallbladder perforation, the operative time was similar between the two groups (59.2 ± 14 *vs* 61.9 ± 12.2 min, p = 0.645), and the authors identified the occurrence of perforation as a risk factor for lengthening of the procedure (Table 6).

**Table 5:** Intra-operative and post-operative outcomes from RCTs included in Metanalysis

Author [ref]	LOS (days)		Conversion Open n of pts (%)		Mean/median Operative time (min)		Bleeding ml/n pts (%)		Perforations n of pts (%)	
	Study	Control	Study	Control	Study	Control	Study	Control	Study	Control
Wetter A.L. [11]	1 (laser)	1.1	2 excluded from the study		56*	97*	0	1 (4.8)	NR	
	1.4 (CUSA)				90*		4 (10.8)			
Tsimoyiannis E.C. [17]	1.4* ± 0.2	1.9* ± 0.5	NA		37* ± 9	45* ± 7	14**	2**	NA	
Sietses C. [18]	2	2	0	0	51* ± 6	57 * ± 14	0 Post-op trans	0 Post-op transf		
Janssen I.M.C. [19]	NR		NR		60** (range 28–186)	65** (range32-180)	NR			
Cenzig Y [20]	2 Pts stayed overnight	8 pts stayed overnight	NR		43* (95% CI 39–48) no cholecystitis 50* (95% CI 41–58) cholecystitis	55*(95% CI 47–64) no cholecystistis 67* (95% CI 59–75) cholecystitis	NR		NR	
Cenzig Y [21]	Day care: 157	Day care: 164	NR		58* (95% CI 53–63)	63 (95% CI 58–68) 74 (95% CI 37–82) Fundus first	12 (95% CI 8–17)	36 (95% CI 26–47) 53 (95% CI 40–67)	19 (26)	39 (51) 37 (46)
Kandil T. [22]	23.44* ± 2.29 (hours)	26.95* ± 8.94 (hours)	0	2 (2.9)	33.21* ± 9.62	51.7* ± 13.79	43.28* + 31.27	83.31* ± 46.23		
El Nakeeb A. [23]	21.87* ± 4.34 (range18–36)	34.54* ± 16.99 (range 18–96)	2 (3.3)	3 (5)	45.17 * ± 10.54 (range 30–70)	69.7* ± 13.01 (range 30–90)	70.13* ± 80.79 (10–400)	133* ± 131.13 (range 10–500)	6(10)	11(18.3)
Redwan A.A. [24]	1** 1.00* ± 0.00 (range 1)	1.5** 1.53* ± 0.51 (range 1–2)	0	0	20**;16.8* ± 6.8 (range 9–30)	45** (44.01* ± 6.47) (range 35–55)	0	1 (1.3)	0	NR but among the causes of IO bile spillage
Mahabaleshwar V. [25]	NR		0	0	27.20*	34.37*	0	0	5 (16.7)	12 (40)
Jain S. K. [26]	1.89* ± 0.56 (range 1–3)	2.52* ± 0.75 (range 2–7)	4 (excluded from the study)	4 (excluded from the study)	50.00* ± 9.356 (range 31–72)	64.70* ± 13.74 (range 42–102)	NR		9	18
Tempè F. [27]	2 Overnight stays	8 Overnight stays	7 excluded from the study		46* ± 13.7	60.8* ± 16.8	NR		NR	
Bulus H. (2013) [11]	NR		NR		NR	NR	NR		NR	
Ramzanali S.A.A [28]	NR		NR		40* ± 4.2	80* ± 17.2	NR		NR	
Catena F. [29]	5.2* ± 0.9	5.4* ± 1.1	1(4.7)	7 (33)	101.3* ± 10.1	106.4* ± 11.3	91.1* ± 11.9	166.6* ± 19.2	NR	
Sista F [30]	3.3* (2–8)	4.1* (2–11)	1 (4)	3 (7)	55.2* (range 39–90)	70.1* (range 51–115)	NR		NR	
Baloch S.H. [31]	NR		3 (7)	2 (4.7)	21.55* (range 12 – 38)	26.63* (range 15 – 44)	0	1 post-op	NR	
Liao G. [32]	3.0* ± 0.4	2.9 * ± 0.4	1 (0.8)	0	54.9* ± 13.1	51.7* ± 9.6	14.2* ± 10.6	13.7* ± 9.1	NR	
Shabbir A. [33]	NR		NR		31.97* ± 4.34	34.05* ± 4.62	NR		12(19.05)	29(46.03)
Ahmed A. [34]	26.31* ± 5.01 hours	28.69* ± 6.16 hours	NR		27.74 * ± 5.37	43.71* ± 5.05	3.13 * ± 1.86	7.14 * ± 3.56	5 (6.9)	19 (26.4)

*Pts* patients, *h* hour, *d* day, *min* minutes, *N* number, *LP* low pressure, *NR*: not reported, *NA* Not available, *ref* reference, *LOS* length of stay, * ±  = mean ± Standard devition, ** = median

**Table 6:** Intra-operative and post-operative outcomes from observational studies

Author [ref]	LOS (days)		Conversion Open n of pts (%)		Operative time (min)		Intra-Bleeding (ml)		Perforation n of pts (%)	
	Study	Control	Study	Control	Study	Comparator	Study	Comparator	Study	Control
Gelmini R. [13]	2 (1–16)	2 (1–12)	1	0	60** (range20–205)	85** (range 45–150)	0	1 post-op	NR	
Zanghì A. [14]	48.15* ± 4.29 hours	49.06* ± 2.94 hours	1	4 (3.3)	35.36 * ± 10.15	55.6* ± 12.10	29.32* ± 14.21	12.41* ± 8.22	3 (6.98)	25 (20.66)
Shulze S. [14]	NR		NR		NR		NR		NR	
Rajinish K [16]	NR		NR		64.3* ± 8.5 (range 49–78)	67.3* ± 9.65 (range 54–90)	NR		4 (20)	6 (30)
Bessa S.S [12]	NR		NR		32.1* ± 7.6 Without perforation 59.2 * ± 14 Wit perforation	36.1* ± 6.20 Without perforation 61.9* ± 12.2 With perforation	NR		10%	30%

*Pts* patients, *h* hour; *d* day, *SD* standard deviation, *N* number, *LP* low pressure, *NR* not reported, ref reference *LOS* length of stay, * = mean; ** = median

### Intra-operative blood loss

Was evaluated in 11 studies (Wetter 1992, Tsimoyiannis 1998, Cengiz 2009, Kandil 2010, El Nakeeeb 2010, Zanghì 2014, Mahabaleshwar 2016, Catena 2014, Liao 2016, Radwan 2010, Ahmed 2019). Generally, blood loss was lower in the HED group. The difference reached a statical significance in six papers (Cenzig 2009, Kandil 2010, El NAkeeb 2010, Zanghì 2014, Catena 2014, Ahmed 2019), whereas in one study, it was not significant (Liao 2016). In two papers, the authors reported no blood loss during surgery (Siestes 2000, Mahabaleshwar 2016). In one paper, the authors reported higher blood loss in the HED group (Tsimoyiannis 1998). In two studies, the number of patients experiencing blood loss who required further interventions was reported instead of the amount of blood loss (Wetter 1992, Redwan 2010). Wetter et al. [10] reported a slightly higher number of patients experiencing an intraoperative blood loss > 10 ml in the CUSA group compared with the electrocautery group (4 *vs* 1); however, bleeding never exceeded 50 ml. In Redwan et al. [24], intra-operative bleeding in the electrocautery group was reported in one case, whereas no bleeding was observed in the HED group. Jannsen et al. [19] reported the need for additional electrocautery in 39 (41%) patients in the HED group.

The occurrence of *intraoperative complications* was described in 10 RCTs (Janssen 2003, Cengiz 2009, Kandil 2010, El Nakeeb 2010, Redwan 2010, Mahabelasheshwar 2011, Ramzanali 2013, Shabbir 2013, Liao 2016, Ahmed 2019). Table 7 and Table 8 summarise the intraoperative findings. The most commonly reported intraoperative complication was gallbladder perforation, with associated bile and/or stone spillage. Two papers (Ramzanali 2013, Shabbir 2016) specifically addressed the difference in gallbladder perforation. The occurrence of gallbladder perforation was significantly lower in the HED group in seven studies (Ahmed 2019, Mahabaleshwar 2011, Janssen 2003, Cengiz 2009, Bessa 2008, Kandil 2010, Shabbir 2016). In contrast, it was lower in the HED group but without statistical significance in two studies (Ramzanali 2013, El Nakeeb 2010). Mahabaleshwar et al. found that there was a 14.23 times greater risk of gallbladder rupture in the presence of complications [25]. Redwan et al. [24] reported the occurrence of intra-operative bile spillage both from the gallbladder and cystic duct. Only in one study did the authors report the occurrence of common bile duct injuries requiring conversion to open surgery in the HED group (Liao 2016). The conversion rate was reported in eight studies (Wetter 1992, Kandil 2010, El Nakeeb 2010, Jain 2011, Tempè 2013, Catena 2014, Sista 2014, Baloch 2016, Liao 2016). In three studies, the converted patients were excluded from the statistical analysis (Wetter 1992, Tempè 2013, Jain 2011). The most common cause of conversion to open surgery was the disrupted anatomy of the Calot’s Triangle (Table 7). The occurrence of gallbladder perforation was lower in two observational studies included in the systematic review (Zanghì 2014, Rajinish 2018) and statically different in one study (Zanghì 2014, *p* < 0.05, 3 vs 25 in HED and Electrocautery group respectively). Among the included observational studies, Bessa et al. [12] reported a significantly higher occurrence of gallbladder perforation in the control group (10% vs 30%; *p* = 0.002) (Table 8).

**Table 7:** Overall numbers and type of intra-operative and post-operative complications in the included RCTs studies

Author (year) [ref]	Complications (n/N)				Technical difficulties n (%) and conversions n (%)
	Intraoperative		Post-operative		
	HED	Control	HED	Control	
Wetter A.L. [10]	0		0		1 (4.8) broken handle in electrosurgical hook 2 (13.3) malfunctioning of the laser 8 (21.6) harmonic vibrations in the CUSA handle
Tsimoyiannis E.C. [17]			1 overall PO complication: 1 collection	5 overall PO complications: 5 collections	
Sietses C. (2000) [18]	0		0		NR
Janssen I.M.C. [19]	15 (16) perforations with bile spillage 3 (3) perforations with stone spillage 39 (41) Additional electrocautery	51 (50) perforations with bile spillage 20 (19) perforations with stone spillage	NR		
Cenzig Y. [20]	0		2 overall PO complications: 1 port site infection 1 hematoma 2 overnight stays for nausea	1 overall PO complication: 1 slippage of clips with bile spillage requiring relaparotomy 7 overnight stays: 4 pain, 2 nausea, 1 combination of pain and nausea	3 conversions in the US group 1 severe cholecystitis 1 choledocholithiasis 1 suspected gallbladder tumour 3 choledocholithiasis/exploration of the bile duct in control group 2 performed laparoscopically 1 converted
Cenzig Y [21]	Gallbladder perforations: 39 (51) conventional group 37 (46) electrocautery fundus-first 19 (26) US fundus-first		4 Minor complications (pleuritis, port-site infection, and deep infection): 1 in the conventional group, 1 in the electrocautery fundus-first 2 in the US fundus-first procedure		NR
Kandil T. [22]	5 (7.1) Bile spillage	13(18.6) Bile spillage	3 (4.2) overall PO complication Pulmonary: 1 (1.4) Port site infection: 1 (1.4) Collection:1 (1.4)	11 (15.71) overall PO complication Pulmonary: 3 (4.3) Port site infection: 4 (5.7) Collection: 2 (2.9) Bile leak: 2 (2.9)	2 conversions (2.9%) one due to unclear anatomy 1 conversion for bleeding in control group
El Nakeeb A. [23]	6 (10) Gallbladder perforations	11 (18.3) Gallbladder perforations 2 bleeding	7 (in 5 pts) overall PO complication Pulmonary infections 2 (3.3) Deterioration of liver function 1 (1.7) Ascetic leak 1 (1.7) Bile leak 1 (1.7) Postoperative collection 3 (5)	15 (in 9 pts) overall PO complication Pulmonary infections 3 (5) Deterioration of liver function 3 (5) Ascetic leak 2 (3.3) Bile leak 2 (3.3) Mild encephalopathy 1 (1.7) Wound infections 3 (5) Postoperative collection 7 (11.7) Incisional hernia 1 (1.7)	3 conversions (5) in the control group (1 unclear anatomy, 2 bleeding) 2 conversions in the US group (unclear anatomy)
Redwan A.A. [24]	8 bile spillage (10)	11 bile spillage (13)		1 bile leak	difficult maneuvering in both groups: 1 due to distended, obstructed bladder in HS 1 acute cholecystitis in the LC
Mahabaleshwar V. [25]	5 (16.7) Gallbladder perforation with bile leak 2 () Gallbladder perforation with stone spillage	12 (40) Gallbladder perforation with bile leak 7 () Gallbladder perforation with stone spillage	3 overall PO complications: 2 port site infections		
Jain S. K. [26]	NR		No incidence of any major complications: bile leak, peritonitis, or bowel injury There was greater fall in hemoglobin (0.53 versus 1.33 g%; P value of .001) and hematocrit (1.59 versus 2.60; P value of .001) when electrocautery was used		8 conversions (4 vs 4) for difficult anatomy of the Calot’s triangle
Tempè F. [27]	NR		2 overnight stays	8 overnight stays 2 in fundus first 1 port-site hematoma 1 bile leakage in CL	
Bulus H. [11]	NR		NR		NR
Ramzanali [28]	2 Gallbladder perforations	3 Gallbladder perforations 4 Stone spillage	NR		NR
Catena F. [29]			4 overall PO complications 1 (4.7) Bile leak Non-surgical morbidity 3 (14) 1 Pneumonia 1 UTI	6 overall PO complications Surgical morbidity 2 () 1 (4.7) Bile leak 1 (4.7) Wound infection Non-surgical morbidity 4 (19) 2 pneumonia UTI	1 (4.7) conversion in the H group for distortion of Calot’s triangle anatomy 7 (33.4) conversions in the EC group:2 for distrsion of the Calot’s triangle anatomy, 3for bleeding and 2 for time exceeding 2 h
Sista F. [30]	NR		NR		NR
Baloch S.H. [31]	NR		1 overall PO complication: 1 bile leak	2 overall PO complications: 1 bleeding 1 bile leak	NR
Liao G. [32]	1 CBD injury	0	2 overall PO complications: 1 pneumonia 1 bile leak	1 overall PO complication: 1 surgical site infection	1 conversion in the US group for CBD
Shabbir A. (2016) [33]	12 (19.5) Gallbladder perforations	29(46.03) Gallbladder perforations	NR		NR
Ahmed A. [34]	5 (6.9) Gallbladder perforations	19 (26.4) Gallbladder perforations	3 overall PO complications: 3 (4.2) fever	12 overall PO complications: 1 (1.4) collection 11 (15.3) fever	NR

*Pts* patients, *ref* reference, *HED* High Energy Device, *NR* Not reported, *NA* Not available, *US* Ultrasonic, *UTI* Urinary Tract infection

**Table 8:** Overall numbers and type of intra-operative and post-operative complications in the included cohort studies

Author (year) [ref]	Complications (n/N)				Technical difficulties *n* (%) and conversions *n* (%)
	Intraoperative		Post-operative		
	HED	Comparator	HED	Comparator	
Gelmini R. [13]			2 overall PO complications: 2 fluid collections	2 overall PO complications 1 hemoperitoneum 1 pleural effusion	1 in US for diffuse peritoneal adhesions (1)
Zanghì A. [14]	3 (6.98) Gallbladder perforations	25 (20.66) Gallbladder perforations	6 overall PO complications: Minor complications 5 (11.62) Bile leaks (observation) 1 (2.43) Abdominal fluid collection 1(2.43) Subclinical increase in pancreatic enzymes 1 (2.43) Urinary retention 1 (0.83) Fever 2 (4.65) 1 major complication (2.43) 1 bleeding requiring laparotomy	6 overall PO complications: Minor complications: 14 (11.57) Bile leaks (observation) 1 (0.83) Abdominal fluid collection 2 (1.65) Subclinical increase in pancreatic enzymes 3 (2.48) Pleural effusion 1 (0.83) Jaundice 1 (0.83) Urinary retention 1 (0.83) Fever 5 (4.13) Major complications: 2(1.65) 1 bile leak 1 ileal perforation	4 conversions in LC (3.3) 1 in the HS group for diffuse peritoneal adhesions
Shulze S. [15]			1 collection	1 readmission for pain	NR
Rajinish K [16]	4(20%)	6(30%)	2(10) SSI	1 (5) collection 3 (15) SSi	**NR**
Bessa S.S [12]	6 (10) Gallbladder perforations	20 (30) Gallbladder perforations	3 overall PO complications: 2 (3.3) Port site infection 1 (1.6) Chest infection	3 overall PO complications: 3 (5) Port site infection 1 (1.6) Chest infection	NR

*Pts* patients, *ref* reference, *HED* High Energy Device, *NR* Not reported; *NA* Not available, *US* Ultrasonic, *PO* post operative, *SSI* Surgical Site Infection

*Post-operative complications* were described in 13, including RCTs (Ahmed 2019, Baloch 2015, Catena 2014, Cengiz 2005, Cengiz 2009, El Nakeeb 2010, Kandil 2010, Liao 2016, Mahabaleshwar 2012, Redwan 2010, Tempè 2013, Tsimoyiannis 1998). The occurrence of post-operative complications was generally higher in the electrocautery group, but it was statically significant only in one study (Ahmed 2019). Post-operative bile leak was reported in four RCTs (Ahmed 2019, Baloch 2015, Catena 2014, El Nakeeb 2010). Kandil et al. distinguished between surgical and general complications, but still, no differences were observed [22]. No statical significance was noted in the occurrence of post-operative complications in the included observational studies. Zanghì et al. [14] divided the outcome into minor and major complications. Among the latter, one haemoperitoneum requiring relaparotomy was described in the HED group, and one ileal perforation and one bile leak were reported in the electrocautery group.

*Length of hospital stay* was described in ten RCTs (Ahmed 2019, Liao 2016, Sista 2014, Jain 2011, Redwan 2010, El Nakeeb 2010, Kandil 2010, Siestes 2000, Tsymoyiannis 1998, Wetter 1992). Two studies reported the need for overnight hospital stays (Cengiz 2005, Tempè 2013). In Cengiz 2009, the number of patients treated with same-day surgery was not statistically different [21]. In Cengiz et al. paper, 2 patients stayed overnight in the HED group and 8 in the Electrocautery group (*p* = 0.036). The reasons for an overnight stay in this group were pain (four patients), nausea (two) and a combination of pain and nausea (two). Two patients stayed in the hospital overnight after the fundus-first dissection because of nausea. [20] The results were similar to Tempè et al. paper (2 vs 8 overnight stays in HED and Electrocautery groups, respectively; *p* = 0036). No reason for hospitalisation was provided. [27]

The results of qualitative analysis for other outcomes are shown in Supplemental Box 2 and Supplemental Box 3.

*The conversion rate* was reported in nine studies (Wetter 1992, Kandil 2010, El Nakeeb 2010, Jain 2011, Tempè 2013, Catena 2014, Sista 2014, Baloch 2010, Liao 2016). Conversion to open surgery was an exclusion criterion in three studies (Wetter 1992, Jain 2011, Tempè 2013). The conversion rate never differed comparing the two groups. The most common cause for conversion was the unclear or distorted anatomy of Calot’s triangle. Among the included observational studies, two reported the occurrence of conversions (Gelmini 2010, Zanghì 2014) due to adhesions.

*Smoke production* None of the included studies directly addressed this outcome. However, two studies (Jansen 2003, Mahabaleshwar 2011) reported the need for lens cleaning during the operation that was not significantly different between the two groups in Jansen et al. [19] but was almost doubled in the electrocautery group compared to the HED group in Mahabaleshwar et al. [25].

### Results of the meta-analysis

The results of the pooled analyses were summarised in the summary of findings table using GRADEPro (https://gradepro.org/cite/gradepro.org.) [36].

**Fig. 2:**
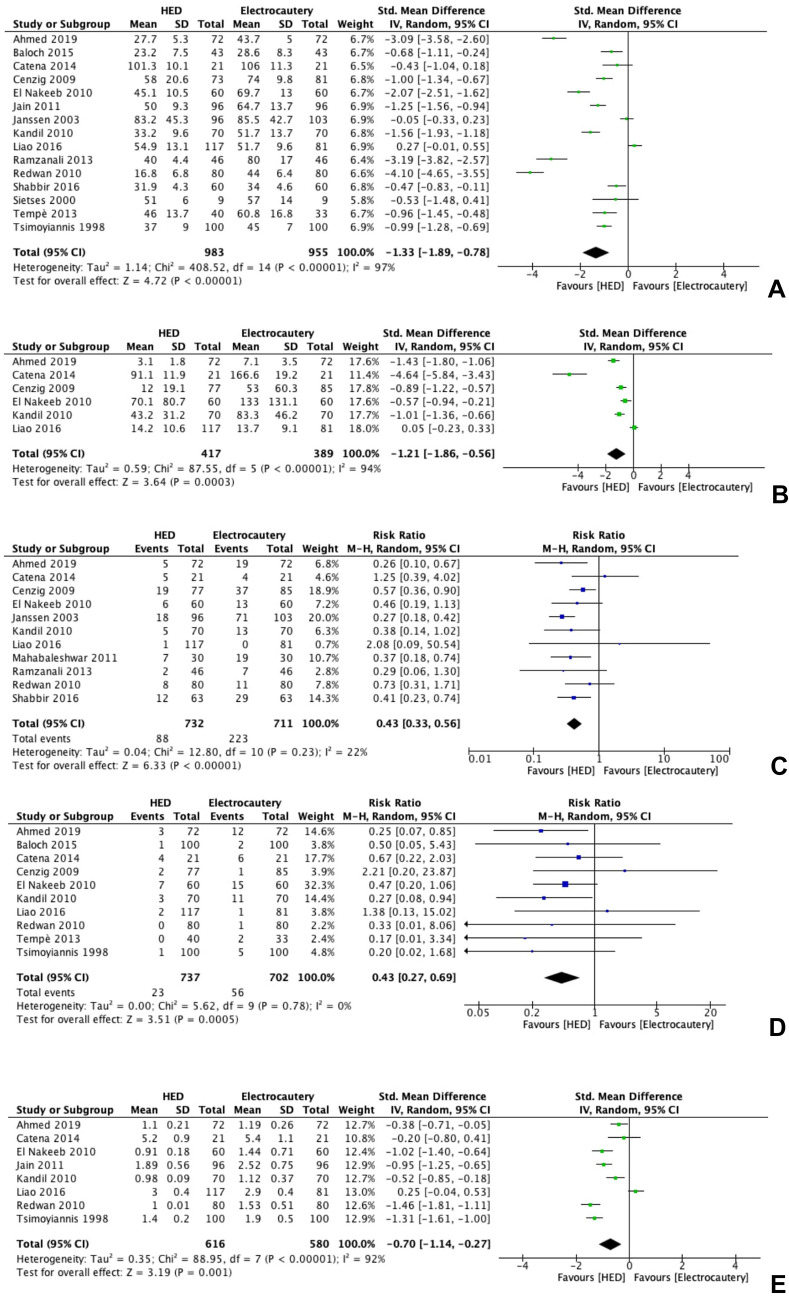
Forest plot originating from the metanalysis of the included RCTs. **A**: operative time; **B**: Intra-operative blood loss; **C**: intra-operative complications; **D**: Post-operative complications; **E**: Length of Hospital stay

*Operative time* was significantly shorter in the HED group than in the electrosurgery group (15 studies, 1938 patients; SMD -1.33; 95% CI -1.89 to 0.78; I2 = 97%, Random-effect) (Fig. 2a).

Two studies explicitly related to cholecystitis. In the sensitivity analysis, six studies with a low risk of bias were included in the meta-analysis (6 studies, 853 patients, SMD -0.80 95% CI -1.53 to -0.07; I2 = 96%, Random-effect). Cengiz et al. paper [20] was excluded from the metanalysis because the overall mean operative time was not provided.

*Intraoperative blood loss* was higher in the electrosurgery group (6 studies, 806 patients; SMD -1.21, 95% CI -1.86 to -0.56; I2 = 94%, Random-effect). (Fig. 2b).

In the sensitivity analysis, five studies were included in the meta-analysis (5 studies, 662 patients, SMD − 1.17; 95% CI − 1.90 to − 0.44; I2 = 94%, Random-effect), showing that intraoperative blood loss was significantly shorter in the HED group (p < 0.00001).

The difference in the incidence of overall *intraoperative complications* between the two groups was not statistically significant (eleven studies, 1443 patients; RR 0.44, 95% CI 0.33 to 0.57; *I*^*2*^ = 22%, Random-effect) (Fig. 2c). The sensitivity analysis of low-risk of bias studies (seven studies, 921 patients, RR 0.44; 95% CI 0.30 to 0.64; *I*^*2*^ = 40%, Random-effect) confirmed that the difference was not statistically significant.

Concerning *intra-operative gallbladder perforations*, the pooled analysis did not show a statistically significant difference between the two study groups (eight studies, 958 patients, RR 0.35; 95% CI 0.27 to 0.46; *I*^*2*^ = 0%, Random-effect). (Supplemental Fig. 3).

The difference in the incidence of *post-operative complications* between the two groups was not statistically significant (twelve studies, 1519 patients; RR 0.46, 95% CI 0.29 to 0.73; *I*^*2*^ = 0%, Random-effect) (Fig. 2d). In the sensitivity analysis, seven studies were included (seven studies, 835 patients, RR 0.55; 95% CI 0.32 to 0.93; *I*^*2*^ = 0%, Random-effect), showing that the difference was not statistically significant. Regarding the occurrence of *post-operative intra-abdominal collections*, the meta-analysis did not show a statically significant difference between the two study groups (four studies, 602 patients, RR 0.37; 95% CI 0.14 to 0.496; *I*^*2*^ = 0%, p = 0.93, Random-effect). (Supplemental Fig. 4).

The *length of hospital stay* was statically shorter in the HED group than in the electrocautery group (eight studies, 1196 patients; SMD − 0.70, 95% CI − 1.14 to − 0.27; *I*^*2*^ = 92%, Random-effect) (Fig. 2e).

The sensitivity analysis of studies at low risk of bias (five studies, 692 patients, SMD − 0.49; 95% CI − 1.01 to − 0.03; *I*^*2*^ = 91%, Random-effect) confirmed the statistically significant difference in favour of the HED group.

### GRADE assessment

**Fig. 3:**
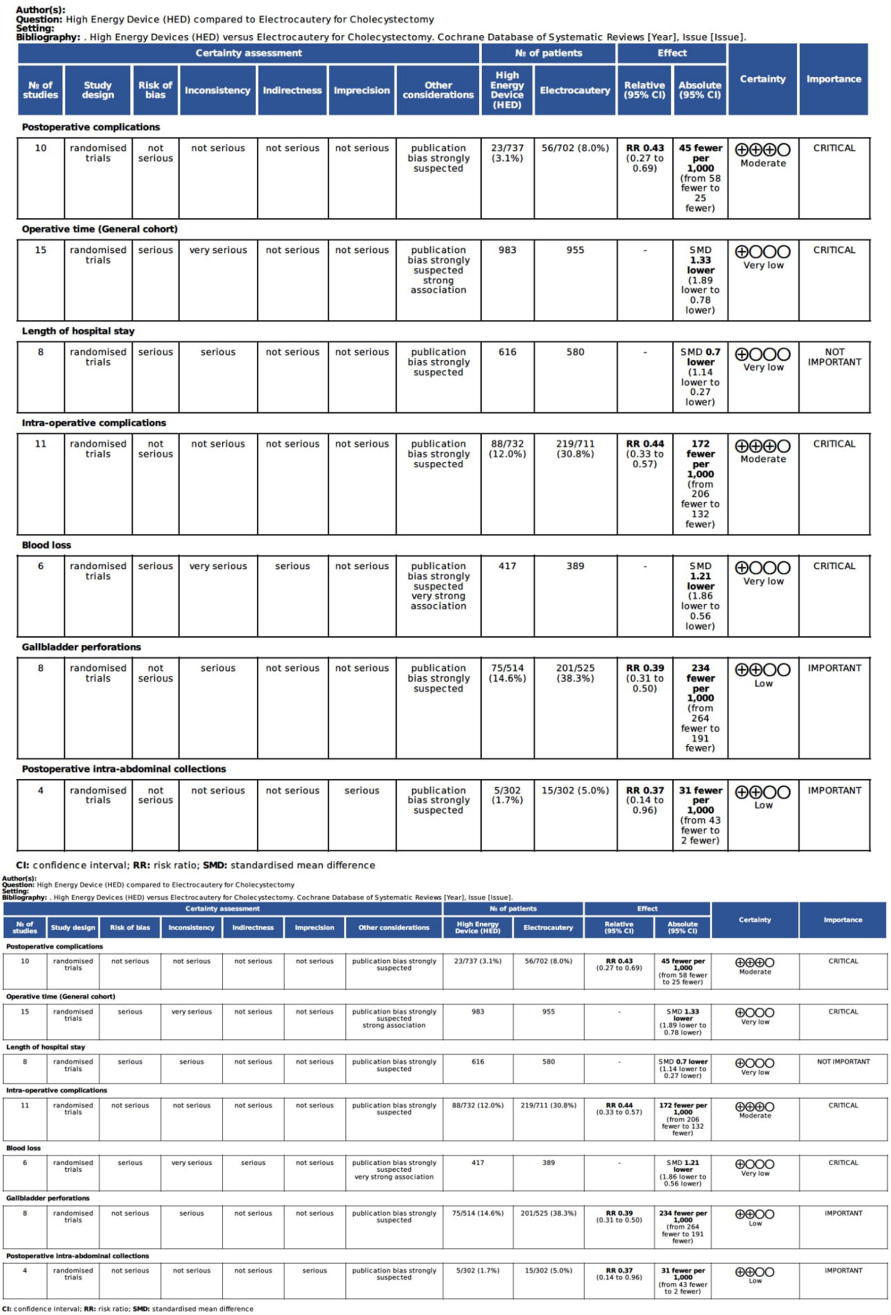
Summary table of the grading of the quality of the evidence

According to the GRADE criteria, the overall quality of evidence was moderate for postoperative complications (critical outcome) and intraoperative complications (critical outcome). The overall quality of evidence was low for operative time (critical outcome), length of hospital stay (important outcome) and blood loss (critical outcome) (Fig. 3).

Risk of Bias assessment for RCTs was reported in Supplemental Table 1 and Supplemental Table 2, and for observational studies in Supplemental Fig. 1. Publication biases are reported in Supplemental Fig. 2.

## Discussion

The interest in HEDs has increased over time, and the reasons behind it are mainly two. The first one is that the last decades have seen an explosion in the type and availability of advanced energy in surgery, undoubtedly fuelled by the minimally invasive “revolution,” which required new devices for tissue dissection and efficient control of larger vessels without suturing [1, 38]. The result was the introduction of clinical use of various devices that apply different energy sources to tissues across all surgical specialities and operative approaches. These technical innovations have enabled advances in minimally invasive surgery, endoscopic interventional techniques and percutaneous procedures for diseases that have greatly enhanced our ability to treat patients. However, this technological boom has also created a dizzying multitude of HED platforms, configurations, generators, cost points and vendors, increasing the complexity and even the potential for injury [38]. As a result, the urge to discipline and implement their use among specialists has increased [1, 38]. Secondly, the diffusion of HEDs had an impact on the economic asset of the healthcare systems. Therefore, when the awareness of financial aspects is becoming predominant, their implementation should be based upon a solid basis demonstrating a higher level of efficiency, proficiency and safety [4, 7]. Furthermore, the increasing discrepancy between technological innovation and technological appraisal, resulting in a wide gap between the introduction of new devices into the market and the adjustment of the legislative system to regulate their implementation in daily clinical practice, is a phenomenon that should be addressed [39].

Only an accurate literature review can answer the continuous need to keep up with the knowledge of the latest available innovations. Three systematic reviews already exist on this topic [5–7]. However, they all revised RCTs comparing ultrasonic devices to monopolar electrocautery in LC. One of them had the primary aim to investigate specifically the safety of US versus conventional metal clips for closure of the cystic duct [7]. Conversely, in the present systematic review and meta-analysis, we performed a comprehensive review of the published literature, including observational and RCTs comparing all advanced energy sources now available to monopolar electrocautery in a specific clinical setting.

Of the included papers, only four investigated other HEDs than ultrasonic devices [10, 11, 15], underlying how US is the energy source more commonly used as an alternative to monopolar energy in LC. This result reflects the conclusions of a previously published survey, where the US was the most used HED in LC [3]. In this survey, the authors concluded HEDs were scarcely used in elective LC. Their explanation for this finding was that in LC, from a technical perspective, the visceral dissection is carried out through a relatively low vascularised plane with only two anatomical structures to seal (cystic artery and cystic duct). Therefore, cholecystectomy is safely performed with a monopolar scalpel and clips without the need for HED in most cases [3]. However, from the present meta-analysis, intra-operative blood loss emerged to be lower in the HED group compared with the electrocautery group. In contrast, the occurrence of intra-operative complications did not statistically differ. Specifically, most of the authors analysing this data showed that gallbladder perforation was reported to occur more often in the electrocautery group, regardless of the experience of the surgeons and the indication for cholecystectomy.

The occurrence of intra- and post-operative complications were the only parameters with a low heterogeneity and were not statistically different between the two study groups. However, the other analysed parameters resulted in a high level of heterogeneity that refrains from drawing definitive conclusions even if operative time, blood loss and length of hospital stay seemed to favour the HED group. So, the question arises as to whether the current body of knowledge could be sufficient to justify the utilisation of a HED in every cholecystectomy or should other factors be considered. Only two studies performed a cost analysis. Undoubtedly, the direct costs of purchasing HED devices are higher than that of monopolar electrocautery. So, the second question arising from this observation is whether these undoubtedly higher direct costs could be adequately balanced by the overall indirect costs linked to the surgical intervention [27]. This topic is too complex to be answered given the lack of available data, and better quality appraisals are needed. As the authors of a previous meta-analysis concluded that HEDs could be superior compared to electrocautery in clinical effectiveness and that further studies focusing on operative time are not needed [6], we may agree that operative time might be not the most important outcome to be investigated in further studies comparing HEDs and electrocautery in LC. Patient-centred outcomes, including post-operative pain, complications, quality of life, and recovery time, should be investigated further in high-quality, powered RCTs. Indeed, the current knowledge is not sufficient to give the possibility to draw conclusive statements on these outcomes.

The knowledge of all the available energy sources to allow their safe implementation in the operating theatre constitutes the rationale for establishing the FUSE (Fundamental Use of Surgical Energy) program. The urge to introduce this type of training was dictated by the high and unacceptable incidence (approximately 1–2 per 1000 operations) of complications derived from the wrong utilisation of these energy sources, without any distinction based on the type of energy [38]. Adverse events due to. energy device use in surgical operating rooms occur regardless of whether the energy source used is.monopolar, bipolar, ultrasound or radiofrequency advanced energy. In 2018, Ha et al. [40] reported that surgeons had a significant knowledge gap in the safe and effective use of surgical energy devices, regardless of surgical speciality and despite what they felt was adequate training. [41, 42]

Our systematic review and meta-analysis demonstrated that the main limitation of the existing literature on HEDs is related to the relatively low quality of available evidence. According to the GRADE criteria, the overall quality of evidence was low for the primary outcome and low to moderate for the further four critical outcomes. Within this context, all the results suggesting any clinical superiority of HEDs over Electrocautery for LC, especially concerning decreased operating times, length of hospitalisation and blood loss, should be interpreted with caution.

## Supplementary Information

Below is the link to the electronic supplementary material.

Supplementary file1 (DOCX 19 KB)

Supplementary file2 (DOCX 16 KB)

Supplementary file3 (DOCX 17 KB)

Supplementary file4 (JPG 174 KB)

Supplementary file5 (JPG 48 KB)

Supplementary file6 (JPG 50 KB)

Supplementary file7 (JPG 66 KB)

Supplementary file8 (JPG 87 KB)

Supplementary file9 (JPG 44 KB)

Supplementary file10 (JPG 91 KB)

Supplementary file11 (DOCX 19 KB)

Supplementary file12 (DOCX 15 KB)
