# Supplementary material for: Use of High Energy Devices (HEDs) versus electrocautery for laparoscopic cholecystectomy: a systematic review and meta-analysis of randomised controlled trials
Source: Surg Endosc. 2023 Apr 19;37(6):4249–69. doi: 10.1007/s00464-023-10060-7 (PMC10235147; doi:10.1007/s00464-023-10060-7)
Supplement: Supplementary file 1 — Supplementary file1 (DOCX 19 KB) [file 464_2023_10060_MOESM1_ESM.docx]

(("high energy device" OR "high energy devices" OR "ultrasonic energy device" OR "ultrasonic energy devices" OR "radiofrequency energy device" OR "radiofrequency energy devices" OR radiofrequency OR ultrasonic OR "hybrid energy" OR ultrasound OR "Ultrasonic coagulating shear" OR "Ultrasonic coagulating shears" OR Ultracision OR Harmonic OR Sonicision OR LigaSure OR Thunderbeat OR Caiman OR Voyant OR Enseal OR Megadyne OR Covidien OR SonoSurg OR ligator OR "vessel sealing technologies" OR "vessel sealing technology" OR "surgical energy device" OR "surgical energy devices" OR "energy device" OR "energy devices" OR "radiofrequency ablation device" OR "radiofrequency ablation devices" OR "ultrasonic scalpel" OR "ultrasonic scalpels") AND ((("monopolar electrosurgery" OR "monopolar scissors" OR "bipolar scissors" OR "bipolar scissor" OR "bipolar electrosurgery" OR "bipolar clamp" OR "bipolar clamps" OR electrocautery OR "monopolar electrocautery" OR "bipolar electrocautery" OR "monopolar electrosurgical" OR electrosurg* OR "monopolar device" OR "monopolar devices" OR "bipolar devices" OR "bipolar device" OR "monopolar system" OR "bipolar system" OR "Electrosurgery"[Majr])))) AND  (("gall-bladder surgery" OR "gallbladder surgery" OR "gall bladder” OR cholecystecto* OR colecystecto*) OR (((("Gallbladder Neoplasms"[Mesh]) OR (gallbladder cancer)) OR ("Cholecystitis"[Mesh])) OR ("Gallbladder Diseases"[Mesh])))

**SUPLLEMENTAL BOX 1.** Primary search strategy
