# Supplementary material for: Use of High Energy Devices (HEDs) versus electrocautery for laparoscopic cholecystectomy: a systematic review and meta-analysis of randomised controlled trials
Source: Surg Endosc. 2023 Apr 19;37(6):4249–69. doi: 10.1007/s00464-023-10060-7 (PMC10235147; doi:10.1007/s00464-023-10060-7)
Supplement: Supplementary file 2 — Supplementary file2 (DOCX 16 KB) [file 464_2023_10060_MOESM2_ESM.docx]

**Post-operative pain score** (assessed using the visual analogue scale -VAS-) and occurrence of nausea and vomiting were reported in six (Cenzig 2005, Kandil 2010, Liao 2016, El Nakeeb 2010, Jain 2014, Tsimoyiannis 1998) and two (Kandil 2010, Liao 2016) studies, respectively. Cenzing et al. [20] reported pain and nausea scores at one, two, four and 24 hours after the operation, and they were always significantly lower in the HED group except at the one-hour interval. Conversely, the Rofecoxib and Paracetamol consumption did not differ statistically between the two groups (p=0.145 and p=0.502, respectively) [20]. In Kandil et al. [22], the incidence of pain was significantly higher in the electrocautery group at 12 hours postoperatively (68.6% vs 51.4%, p=0.03), and VAS scores were significantly lower in the HED group at 12 and 24 hours intervals, p=0.0001). However, the difference was not significant at 48 hours and 1 week postoperatively [22]. Similarly, in Jain et al. [26], the pain scores at any time (day 0 and day 1) and total analgesic consumption differ statistically in favour of the HED group. In El Nakeeb et al. [26], post-operative pain was significantly more severe in the electrocautery group at the 1-day interval (p=0.001), but the difference was not significant after 1 week. Redwan et al. [23] reported that post-operative pain occurred more frequently in the electrocautery group, with an associated higher need for analgesic consumption. Among the included observational studies, only Rajinish et al. reported the VAS scores for pain and overall analgesic consumption, but they were similar in the two groups [16].

**SUPPLEMENTARY BOX 2**. Results of the qualitative analysis for pain
