# Supplementary material for: Use of High Energy Devices (HEDs) versus electrocautery for laparoscopic cholecystectomy: a systematic review and meta-analysis of randomised controlled trials
Source: Surg Endosc. 2023 Apr 19;37(6):4249–69. doi: 10.1007/s00464-023-10060-7 (PMC10235147; doi:10.1007/s00464-023-10060-7)
Supplement: Supplementary file 3 — Supplementary file3 (DOCX 17 KB) [file 464_2023_10060_MOESM3_ESM.docx]

**Cost analysis** was performed in two studies (Tempè 2013, Bulus 201). Tempè et al. performed both a direct and indirect cost analysis [27]. The direct analysis included per-patient costs for blood samples, X-rays, and drugs, as well as costs that are not possible to specify individually, such as costs for personnel, equipment, facilities, heating, cleaning, and buildings, as well as costs for anaesthesia and surgery in addition to the costs for ultrasonic shears. Loss of productivity for working was considered an indirect cost. The authors reported that the direct cost was 1,190 SEK lower with the ultrasonic fundus-first technique than with a conventional method [27]. The total costs, also accounting for sick leave, were 5,370 SEK lower with the fundus-first technique using ultrasonic dissection [27]. Conversely, Bulus et al. [11] reported an additional per-case cost of 100 dollars and 45 dollars, respectively, in the US HED and RF groups compared with electrocautery.

**Other outcomes** Three studies (Cenzig 2005, Cenzig 2009, Tempè 2013) reported that sick leave was significantly shorter in the US group. In the first paper, the authors performed a multivariate analysis demonstrating that the ultrasonic fundus-first dissection was an independent predictor of same-day discharge from the hospital and of shorter sick leave compared to electrocautery [20].

Tempè et al. [27] reported that, for both employed and unemployed patients, sick leave and time to full recovery were approximately three days shorter with the fundus-first method using ultrasonic dissection than with conventional cholecystectomy performed with electrocautery and US dissection [27].

Additionally, in Wetter et al. [10], the mean time to return to work was 7.9 days in the electrocautery group and 11.6 and 8 days in the laser and CUSA groups, respectively.

**SUPPLEMENTARY BOX 3**. Results of the qualitative analysis for cost analysis and other outcome
