# Supplementary figures and images for: Use of High Energy Devices (HEDs) versus electrocautery for laparoscopic cholecystectomy: a systematic review and meta-analysis of randomised controlled trials
Source: Surg Endosc. 2023 Apr 19;37(6):4249–69. doi: 10.1007/s00464-023-10060-7 (PMC10235147; doi:10.1007/s00464-023-10060-7)

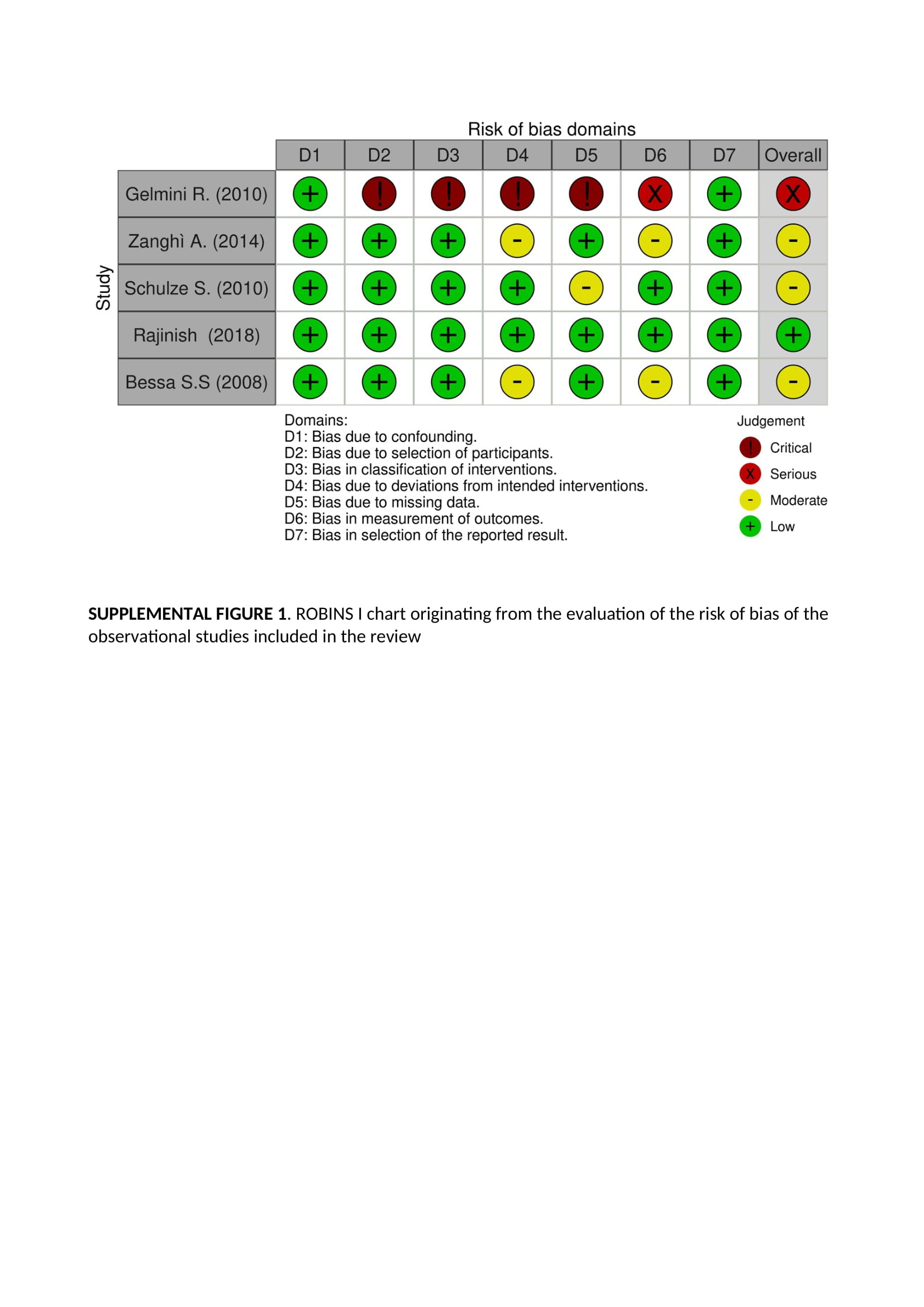

Supplement: Supplementary file 4 — Supplementary file4 (JPG 174 KB) [file 464_2023_10060_MOESM4_ESM.jpg]

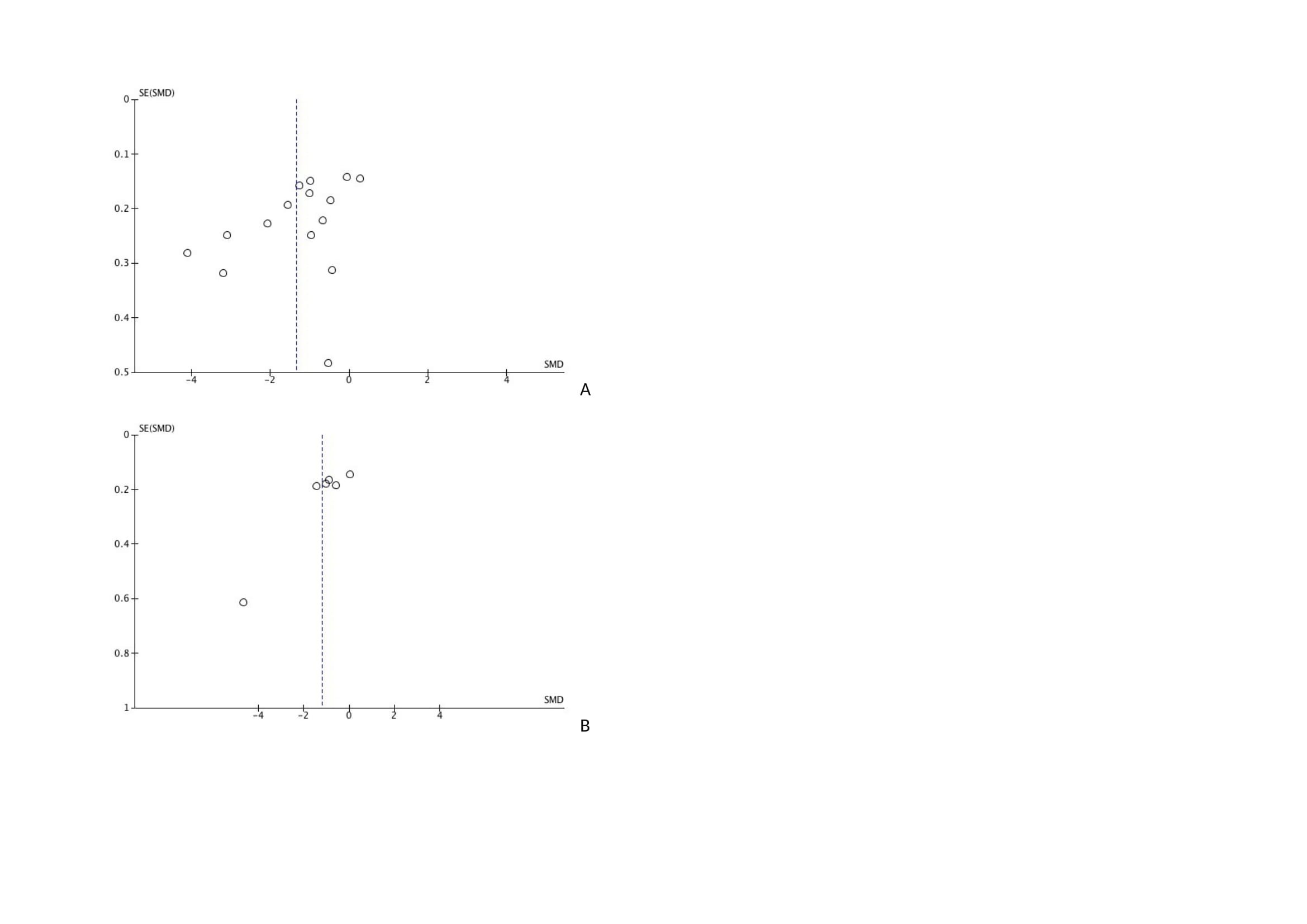

Supplement: Supplementary file 5 — Supplementary file5 (JPG 48 KB) [file 464_2023_10060_MOESM5_ESM.jpg]

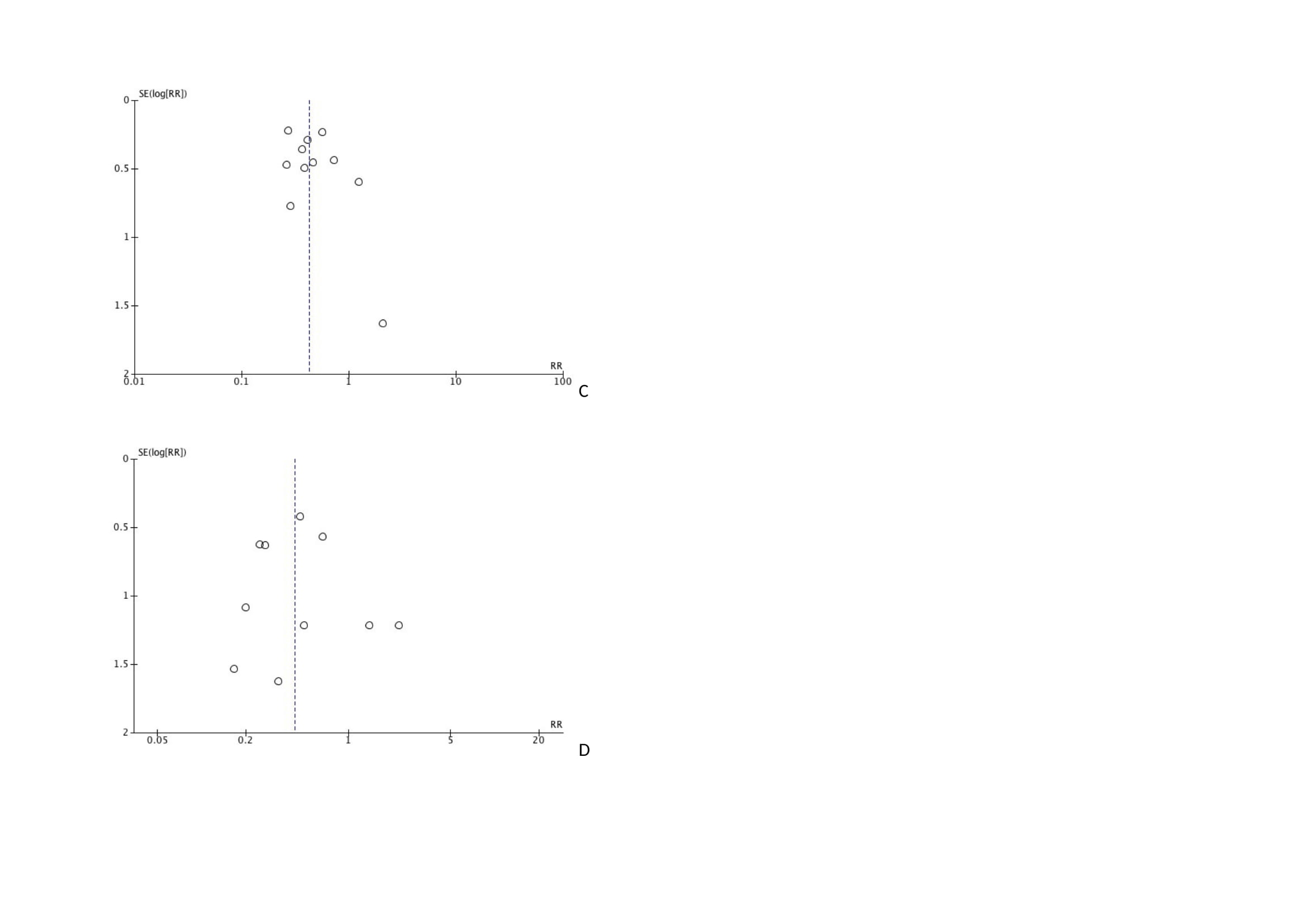

Supplement: Supplementary file 6 — Supplementary file6 (JPG 50 KB) [file 464_2023_10060_MOESM6_ESM.jpg]

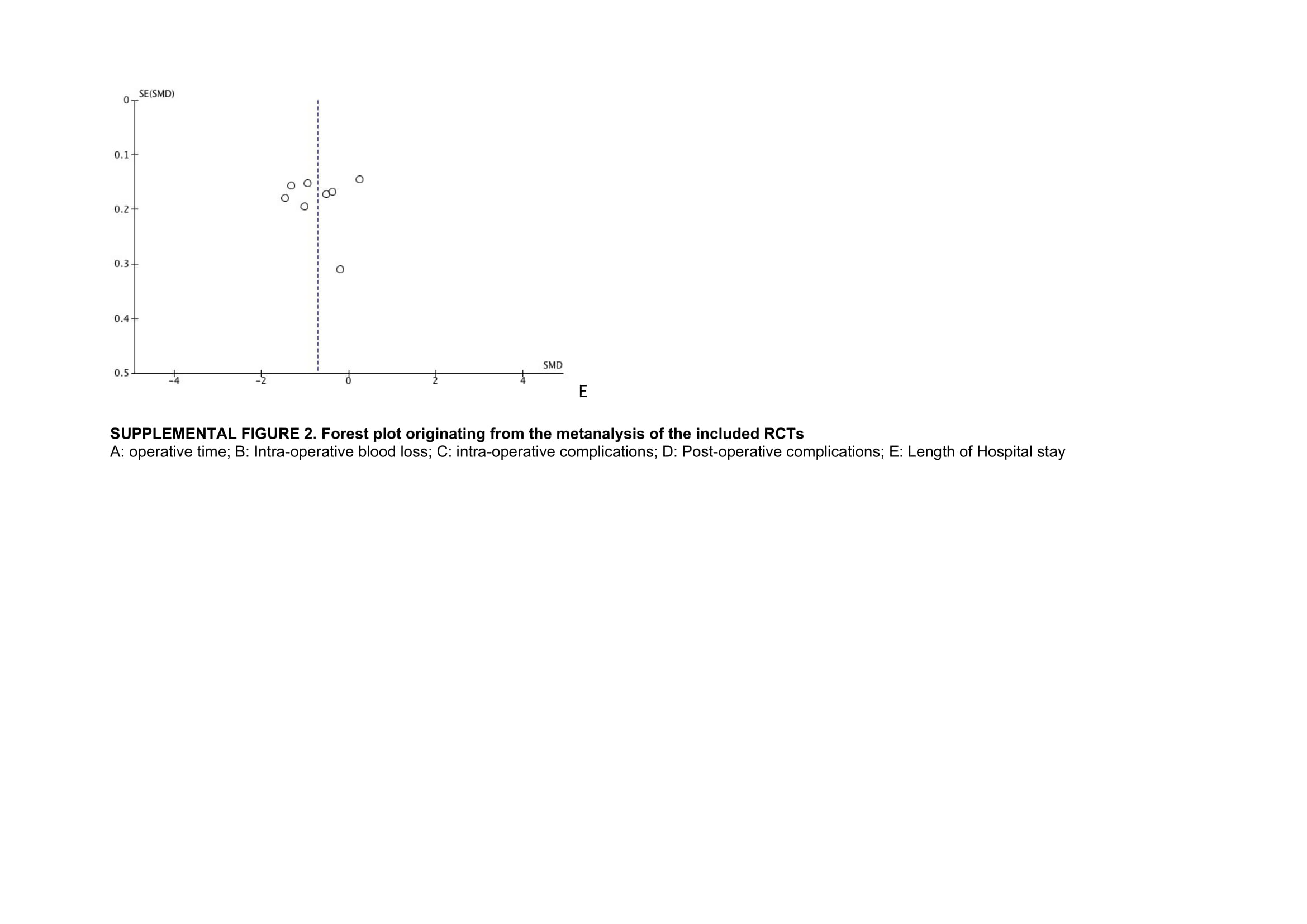

Supplement: Supplementary file 7 — Supplementary file7 (JPG 66 KB) [file 464_2023_10060_MOESM7_ESM.jpg]

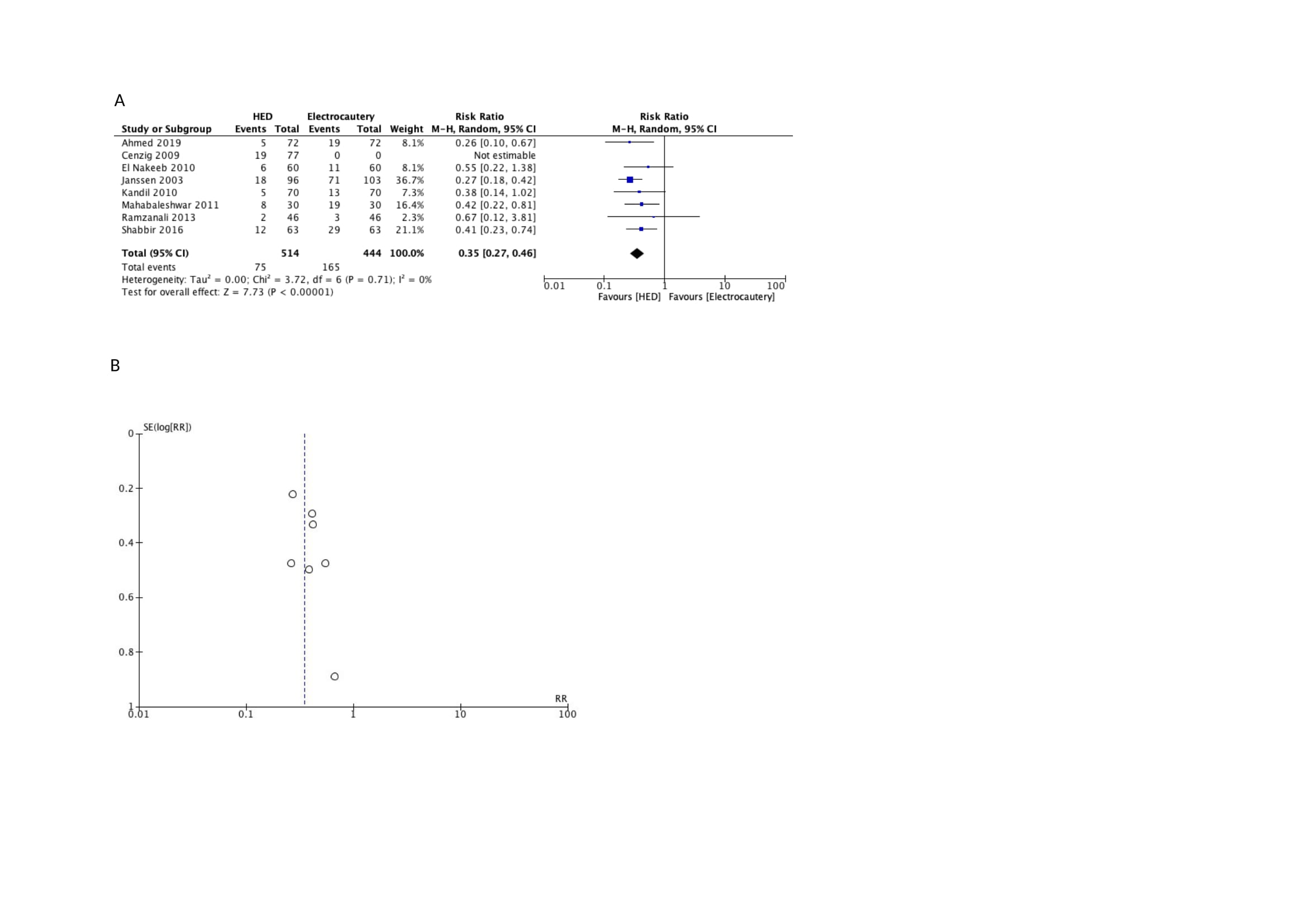

Supplement: Supplementary file 8 — Supplementary file8 (JPG 87 KB) [file 464_2023_10060_MOESM8_ESM.jpg]

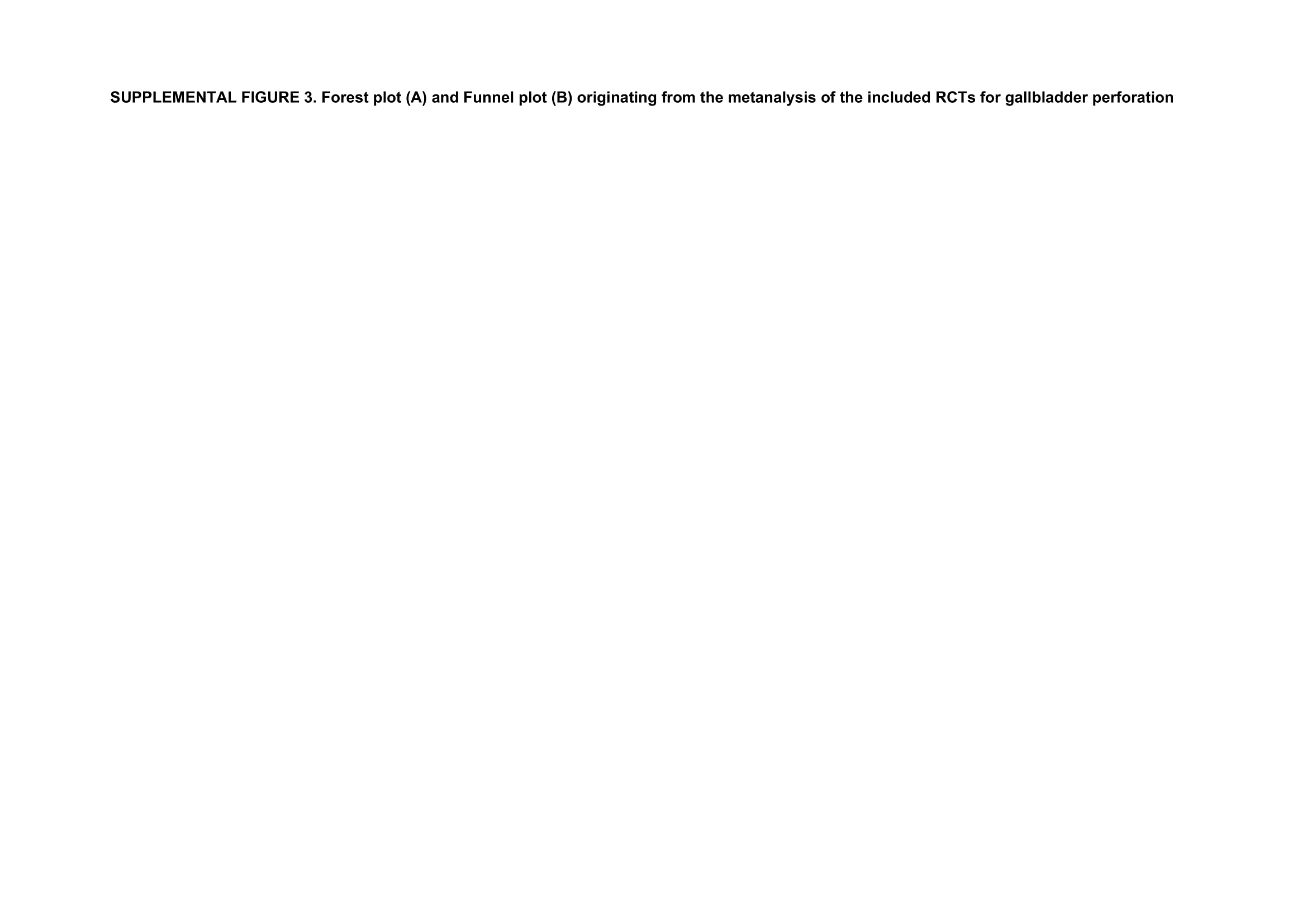

Supplement: Supplementary file 9 — Supplementary file9 (JPG 44 KB) [file 464_2023_10060_MOESM9_ESM.jpg]

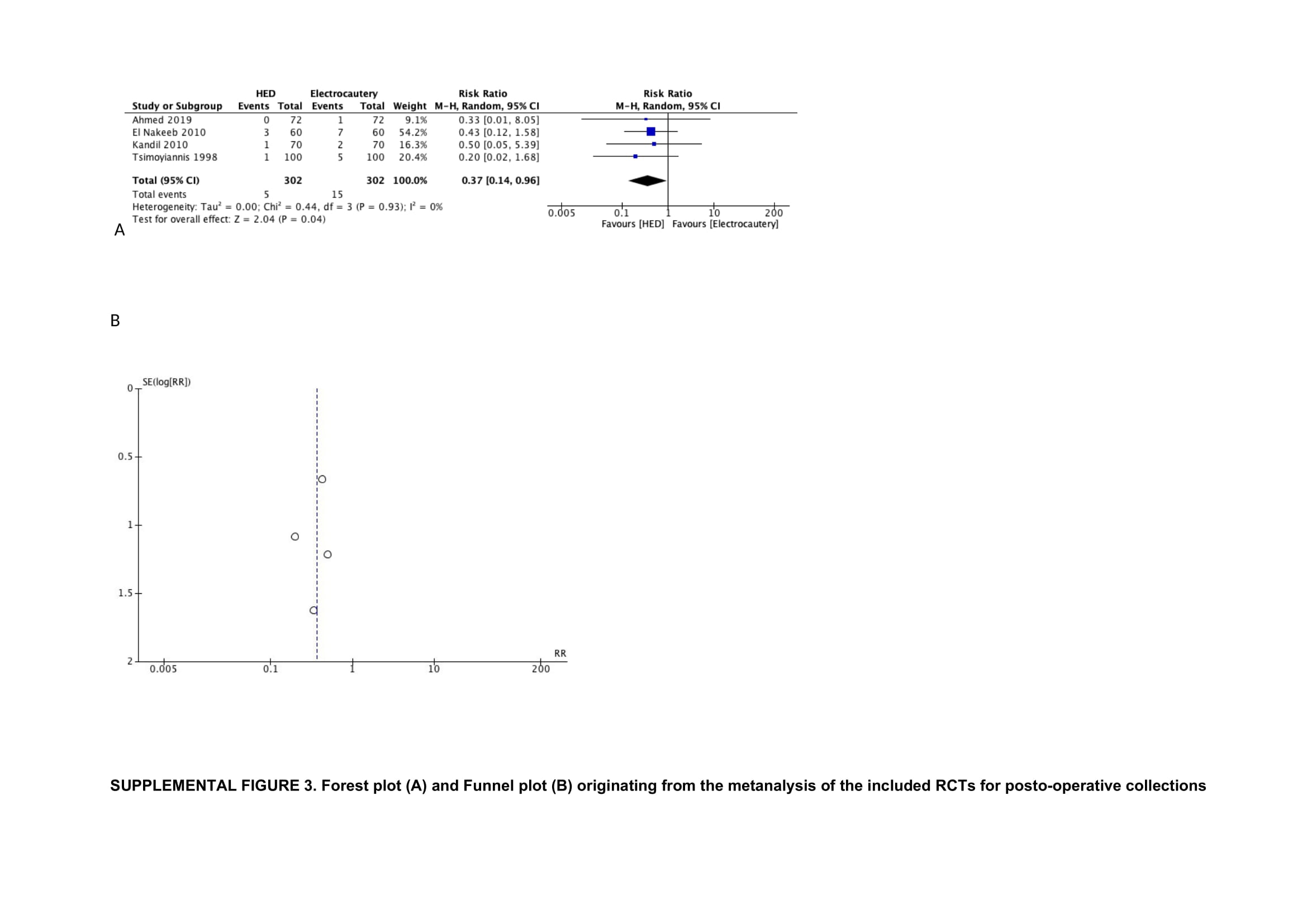

Supplement: Supplementary file 10 — Supplementary file10 (JPG 91 KB) [file 464_2023_10060_MOESM10_ESM.jpg]
