# Supplementary material for: Use of High Energy Devices (HEDs) versus electrocautery for laparoscopic cholecystectomy: a systematic review and meta-analysis of randomised controlled trials
Source: Surg Endosc. 2023 Apr 19;37(6):4249–69. doi: 10.1007/s00464-023-10060-7 (PMC10235147; doi:10.1007/s00464-023-10060-7)
Supplement: Supplementary file 11 — Supplementary file11 (DOCX 19 KB) [file 464_2023_10060_MOESM11_ESM.docx]

| Unique ID | Study ID | Experimental | Comparator | Outcome | D1a | D1b | D2 | D3 | D4 | D5 | Overall |
| --- | --- | --- | --- | --- | --- | --- | --- | --- | --- | --- | --- |
| Wetter A.L. (1992) | 10 | CUSA/LASER | Electrocautery | OT | SC | SC | LR | LR | LR | SC | SC |
| Tsimoyiannis E.C. (1998) | 17 | US | Electrocautery | OT | SC | HR | HR | HR | HR | HR | HR |
| Sietses C. (2000) | 18 | US | Electrocautery | OT | SC | LR | SC | LR | HR | LR | SC |
| Janssen I.M.C. (2003) | 19 | US | Electrocautery | OT | LR | LR | LR | LR | LR | LR | LR |
| Cenzig Y(2005) |  | US | Electrocautery | OT | LR | LR | LR | LR | LR | LR | LR |
| Cenzig Y(2005) | 20 | US | Electrocautery | OT | LR | LR | LR | LR | LR | LR | LR |
| Cenzig Y (2009) | 21 | US | Electrocautery | OT | LR | LR | LR | LR | LR | LR | LR |
| Kandil T. (2010) | 22 | US | Electrocautery | OT | LR | LR | LR | LR | LR | LR | LR |
| El Nakeeb A. (2010) | 23 | US | Electrocautery | OT | LR | LR | LR | LR | LR | LR | LR |
| Redwan A.A. (2010) | 24 | US | Electrocautery | OT | SC | LR | LR | LR | LR | SC | SC |
| Mahabaleshwar (2011) | 25 | US | Electrocautery | OT | LR | LR | LR | LR | LR | LR | LR |
| Jain S. K. (2011) | 26 | US | Electrocautery | OT | SC | LR | LR | LR | LR | SC | SC |
| Tempè F. (2013) | 27 | US | Electrocautery | OT | SC | LR | LR | LR | LR | SC | SC |
| Bulus H. (2013) | 11 | US/RF | Electrocautery | OT | LR | LR | LR | LR | LR | LR | LR |
| Ramzanali (2013) | 28 | US | Electrocautery | OT | SC | LR | LR | LR | LR | SC | SC |
| Catena F. (2014) | 29 | US | Electrocautery | OT | LR | LR | LR | LR | LR | LR | LR |
| Sista F (2014) | 30 | US | Electrocautery | OT | SC | LR | SC | LR | LR | SC | SC |
| Baloch S.H. (2015) | 31 | US | Electrocautery | OT | SC | LR | HR | HR | HR | SC | HR |
| Liao G. (2016) | 32 | US | Electrocautery | OT | LR | LR | LR | LR | LR | LR | LR |
| Shabbir A. (2016) | 33 | US | Electrocautery | OT | SC | SC | HR | HR | HR | SC | HR |
| Ahmed (2019) | 34 | US | Electrocautery | OT | SC | SC | HR | HR | HR | SC | HR |

**SUPPLEMENTAL TABLE 1**. RoB-2 summary table for the included RCTs
