# Supplementary material for: Use of High Energy Devices (HEDs) versus electrocautery for laparoscopic cholecystectomy: a systematic review and meta-analysis of randomised controlled trials
Source: Surg Endosc. 2023 Apr 19;37(6):4249–69. doi: 10.1007/s00464-023-10060-7 (PMC10235147; doi:10.1007/s00464-023-10060-7)
Supplement: Supplementary file 12 — Supplementary file12 (DOCX 15 KB) [file 464_2023_10060_MOESM12_ESM.docx]

| Study | D1 | D2 | D3 | D4 | D5 | D6 | D7 | Overall |
| --- | --- | --- | --- | --- | --- | --- | --- | --- |
| Gelmini R. (2010) | Low | Critical | Critical | Critical | Critical | Serious | Low | Serious |
| Zanghì A. (2014) | Low | Low | Low | Moderate | Low | Moderate | Low | Moderate |
| Schulze S. (2010) | Low | Low | Low | Low | Moderate | Low | Low | Moderate |
| Rajinish (2018) | Low | Low | Low | Low | Low | Low | Low | Low |
| Bessa S.S (2008) | Low | Low | Low | Moderate | Low | Moderate | Low | Moderate |

**SUPPLEMENTAL Table 2**. ROBINS I chart originating from the evaluation of the risk of bias of the observational studies included in the review
